# Genetic Diversity of HPV 16 and HPV 18 Based on Partial Long Control Region in Iranian Women

**DOI:** 10.1155/2022/4759871

**Published:** 2022-01-25

**Authors:** Mina Mobini Kesheh, Mohadeseh Barazandeh, Amir Kaffashi, Mohammad Kazem Shahkarami, Seyed Alireza Nadji

**Affiliations:** ^1^Department of Virology, School of Medicine, Iran University of Medical Sciences, Tehran, Iran; ^2^Microbiology Department, Qom Islamic Azad University, Qom, Iran; ^3^Razi Vaccine and Serum Research Institute, Agricultural Research, Education and Extension Organization (AREEO), Karaj, Iran; ^4^Virology Research Center, National Research, Institute of Tuberculosis and Lung Disease (NRITLD), Shahid Beheshti University of Medical Sciences, Masih Daneshvari Hospital, Daar-abad, Niavaran, Tehran, Iran

## Abstract

**Background:**

Human papillomavirus **(**HPV) 16 and HPV 18 account for 75% of all cervical cancers. The L1 gene, encoding the major surface protein (MSP), is used to classify HPV types (lineages and sublineages), genotypes, and intratypic variants. Therefore, this study aimed to investigate the lineages, sublineages, genetic variabilities, and mutation effects on transcription factor binding sites by using partial sequences of the HPV 16 and HPV 18 long control regions (LCRs) in these samples.

**Materials and Methods:**

After DNA isolation from 56 positive samples, the LCR of HPV 16 and HPV 18 were amplified using specific primers, and phylogenetic trees were drawn through MEGA X. Compared to the reference sequences, single nucleotide polymorphisms (SNPs) were identified. The transcription binding sites were also evaluated using the online PROMO database.

**Results:**

The LCRs of 52 samples were successfully sequenced. Overall, 81.58% of all HPV 16 variants belonged to the D1 sublineage, followed by A4 (13.16%), A1 (2.63%), and C1 (2.63%) sublineages. All HPV 18 isolates belonged to A sublineage, 92.85% to A3 sublineage, and 7.15% to A4 sublineage. Out of 27 SNPs in the HPV 16 LCR, A7382T, T7384G, C7387T, C7393G, A7431G, T7448C, and C7783A were HPV 16-specific. Also, among 14 SNPs in the HPV 18 LCR, C7577A and A7943T were not previously reported. An insertion (C) between 7432 and 7433 positions was identified in all studied HPV 16 variants. Besides, most of the HPV 16 mutations were embedded in the YY1, TFIID, Oct-2, and NF-1 binding sites, while c-Fos and MBF1, as the most common binding sites, were affected by HPV 18 LCR mutations.

**Conclusion:**

The present results showed that D1 and A3 were the dominant sublineages of HPV 16 and HPV 18, respectively. Therefore, women infected with these variants need to be examined in further longitudinal studies to obtain more information about the oncogenic potential of these dominant variants in Iran. Besides, YY1, TFIID, Oct-2, NF-1, c-Fos, and MBF1 were the most frequent binding sites, which were influenced by the mutations.

## 1. Introduction

Human papillomavirus (HPV) 16 and HPV 18 are well-known viruses, responsible for cervical cancer and associated with some other cancers [1]. HPV 16 and HPV 18 account for 75% of all cervical cancers [2]. HPV 16 variants are classified into four phylogenetic lineages of A, B, C, and D, each with four sublineages of A1–A4 (European and Asian), B1–B4 (African 1), C1–C4 (African 2), and D1–D3 (Asian-American and North American) [3, 4].

Moreover, three major phylogenetic lineages have been identified for HPV 18: A (European and Asian-Amerindian), B, and C (African). Overall, lineages A and B each have four sublineages [5]. While HPV 16 variants differ in terms of oncogenic potency (A3, A4, and D sublineages are associated with a high risk of cervical cancer) [3, 6], there is inconsistent information on HPV 18. Also, in the latest study on HPV 18 variants, no preferred cervical cancer risk was observed among HPV 18 variants [5].

Different genome segments have been used to classify HPV 16 and HPV 18 into lineages, including long control region (LCR), E6, and L1 [7–11]. The LCR contains important regions in viral transcription and replication, including the enhancer, E2 binding, and origin of replication (ori) sites [12]. Some nucleotide changes are responsible for the increased transcription activity in some HPV 16 variants [13].

Due to the importance of LCR in the viral life cycle and reports of mutations in some nucleotide positions of the LCR, causing changes in the viral oncogenic potency (with a potentially important role in viral pathogenicity) [14, 15], this study aimed to identify variants in Iranian women with normal Pap smear results, according to the partial sequences of the LCR, containing the enhancer and E2 binding sites for both HPV genotypes. Also, by using the PROMO database, the effects of single nucleotide polymorphisms (SNPs) on transcription binding sites in the LCRs were evaluated.

## 2. Materials and Methods

### 2.1. Clinical Samples

56 samples were used in this study. Indeed, a total of 2,756 ThinPrep samples were collected from women (age range: 16–48 years) in 12 provinces of Iran for the national HPV screening and genotyping program. The participants were referred to the papillomavirus reference laboratory of Masih Daneshvari Hospital Virology Research Center, Tehran, Iran [16]. Out of 6.1% (167/2,756) of samples that were HPV DNA positive, 24.6% (41/167) and 9.0% (15/167) were positive for HPV 16 and HPV 18, respectively, and enrolled in the study.

### 2.2. HPV 16 and HPV 18 LCR Amplification

DNA was isolated from ThinPrep samples using the High Pure Viral Nucleic Acid Kit (Roche, Germany), according to the manufacturer's instructions. The target regions of HPV 16 and HPV 18 LCRs (a 530-bp region with nucleotides 7,336 to 7,866 and GenBank Accession NC_001526.4 for HPV 16; and a 555-bp region with nucleotides 7465 to 58 and GenBank Accession NC_001357.1 for HPV 18) were amplified by nested polymerase chain reaction (PCR) (for HPV 16) and conventional PCR (for HPV 18). The HPV 16 primers included 5′CTTGTGTAACTATTGTGTCATGC3′ and 5′AGTTGCTTGTAAATGTGTAACCC3 as outer primers and 5′CAACACCTACTAATTGTGTTGTGG3′ and 5′AAATCGGTTTGCACACACCCATGT3′ as inner primers.

The profile PCR reaction was set up in 95°C for 300 s, 95°C for 60 s, 55°C for 60 s, 72°C for 90 s, and final extension in 72°C for 300 s for 35 cycles with outer primers, which led to the production of 600-nucleotide amplimers. The same thermocycler profile was used with inner primers to amplify the final products (530 nucleotides). The HPV 18 primers included 5′TCGGTTGCCTTTGGCTTAT3′ and 5′TCCGTGCACAGATCAGGTAG3'. Similar to HPV 16, a PCR program was performed for HPV 18. The *β*-globin gene was also used to evaluate the sample quality. Amplification of PCR products was confirmed by 1.5% agarose gel electrophoresis.

### 2.3. Sequence Alignment and Phylogenetic Analysis

The partial sequences of the HPV 16 and HPV 18 LCRs were analyzed in BioEdit software [17] and blasted using MEGA X software with reference sequences [18]. Phylogenetic trees were constructed using the neighbor-joining method, Kimura 2-parameter method, and bootstrapping (1000 replicates) in MEGA X software. Overall, 37 complete genome sequences were used as the reference for both viruses.

### 2.4. Identification of SNPs in HPV 16 and HPV 18 LCRs and Evaluation of Transcription Binding Sites

After sequencing, to identify SNPs, the studied HPV 16 and HPV 18 LCR sequences were aligned with the reference sequences (GenBank NC_001526.4 and NC_001357.1, respectively) using MEGA X software. Besides, the online PROMO database was used to evaluate the transcription binding sites [19].

## 3. Results

### 3.1. Successful Amplification

Among 56 DNA-positive samples for both HPV genotypes, two samples of each virus (HPV 16 and HPV 18) were excluded due to poor amplification quality. In the remaining samples (*n* = 52), a 530-nucleotide genotype (7,336 to 7,865 for HPV-16) and a 555-nucleotide genotype (7,465 to 63 for HPV-18) from LCRs were successfully amplified and sequenced.

#### 3.1.1. Tree Construction and Distribution of HPV 16 and HPV 18 Sublineages in Iran

**Figure 1:**
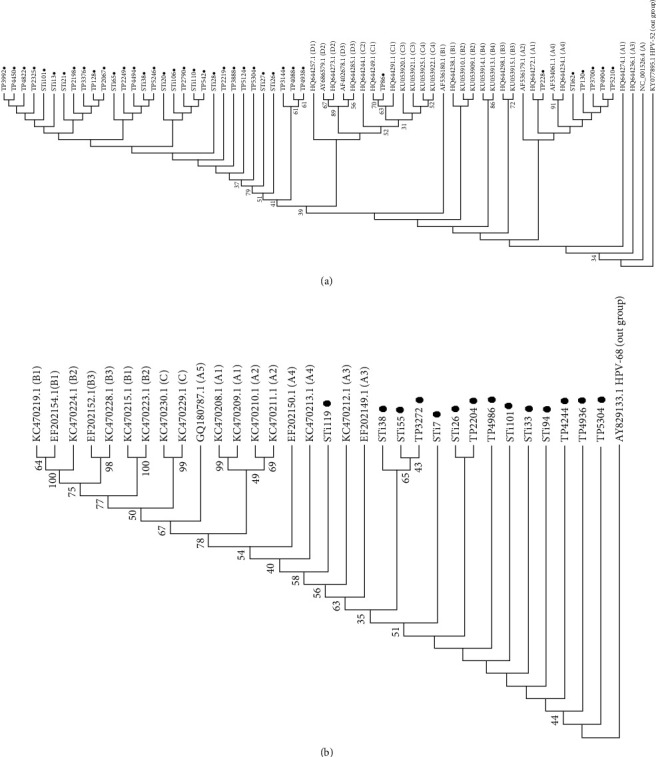
The phylogenetic trees based on the HPV-16 (a) and HPV-18 (b). LCR was constructed using the neighbor-joining method, Kimura 2-parameter method, and Bootstrap 1000 by means of MEGA X. In branches, the values lower than 30% are hidden and samples with small black circles were understudied. HPV-16 and-18 LCR partial sequences of Iranian isolates obtained from this study were recorded in GenBank with accession numbers KF447488-KF447525 and MZ868727-MZ868740, respectively.

The studied HPV-16 variants mainly belonged to D lineage (D1 sublineage), which accounted for 81.58% of all HPV 16-positive samples, followed by A4 (13.16%), A1 (2.63%), and C1 (2.63%). All HPV 18 isolates belonged to A lineage (92.85% to A3 sublineage and 7.15% to A4 sublineage) (Figure 1). The sublineages were identified based on 0.5–1.0% differences between isolate genomes, calculated in MEGA X software. Although the primary samples were collected from 12 provinces of Iran, HPV 16 and HPV 18 DNA-positive samples were found in 11 and four provinces, respectively. The HPV16 C1, HPV16 A1, and HPV18 A3 variants were only found in Ardabil, Kurdistan, and Tehran provinces, respectively (Tables 1 and 2).

### 3.2. Single Nucleotide Polymorphisms (SNPs)

Out of 41 SNPs found in this study, 27 were in the HPV 16 LCR and 14 in the HPV 18 LCR (Tables 1 and 2). Seven SNPs were specific to the sequences of HPV 16, and two SNPs were newly detected in the studied HPV 18 samples (A7382T, T7384G, C7387T, C7393G, A7431G, T7448C, and C7783A in the HPV16 LCR and C7577A and A7943T in the HPV18 LCR). Moreover, an insertion (C) between positions 7432 and 7433 was identified in all studied HPV 16 variants. No insertion or deletion was found in the HPV 18 LCR.

### 3.3. Transcription Binding Sites

Thirteen transcription factors (TFIID, TBP, c-Fos, TEF-1, NF-1, HMGI, RXR*α*, c-Jun, MBF1, C/EBP*α*, AP-1, POU2F1 [Oct1], and E2F) are able to bind to the target region in the HPV 18 LCR, and seven factors (YY1, TFIID, ROX1, POU2F1/2 [Oct1/2], AP-1, E2F, and NF-1) could bind to the HPV 16 LCR. Compared to the HPV 18 reference, except for STi119 isolate, C7486T led to the emergence of binding sites for c-Fos and MBF1 in 93.0% of the samples. Also, in 100% of the samples, a T7595C mutation corresponded to the disappearance of binding sites of TFIID and TBP transcription factors. In the HPV 16 LCR, the most common mutations were embedded in the YY1, TFIID, Oct-2, and NF-1 binding sites (Supplementary Tables 1(a) and 1(b)).

The nucleotide positions of detected mutations are written vertically across the top and are indicated by the corresponding nucleotide letter. The absence of variations relative to the prototypes is represented by dashes. Nucleotide changes in black boxes, in different studies were reported, led to increase in p97 promoter activity and, consequently, increase in the expression of oncogenes E6/E7 in D lineage variants.

HPV-16 ref is NC_001526.1 in Table 1, and HPV-18 ref is NC_001357.1 in Table 2.

## 4. Discussion

HPV 16 and HPV 18 are the most prevalent HPV genotypes in women with normal Pap smear and cancerous samples [20, 21]. Although the burden of HPV 16 and HPV 18 has reduced following vaccination, their potential contribution to cancer progression remains unchanged. One explanation for this finding may be the mutations in different parts of the viral genome, especially along the LCR as the main regulatory region [16, 17].

According to different studies in Iran, it seems that the abundance of HPV 16 is higher than other types of HPV among Iranian women with either normal Pap smear or cancerous samples; however, HPV 18, as the second most dominant genotype in cervical cancer, may not be observed frequently in normal samples [1, 20]. Since both viruses have different variants, many studies have shown that some HPV 16 variants, especially non-A variants, play a critical role in the development of an invasive form of cervical cancer. However, findings regarding the importance of HPV 18 variants in cervical cancer are incompatible [3, 5, 22].

In this study, the common variants of HPV 16 and HPV 18 were investigated. Generally, different regions and gene targets were selected to classify the intratypic HPV 16 and HPV 18 variants. Given the importance of LCRs in viral replication and transcription, especially regions with viral and cellular transcription binding sites, they were selected for this study. In the current study, D1 and A4 sublineages were found as the main HPV 16 variants, which is consistent with a previous study by Vaezi et al. [11]. Commonly, D1 variants are found in Southern Asia, sub-Saharan Africa, Europe, and North America [5].

Although the sample size was small for HPV 18, A3 was the predominant variant, followed by A4. This finding is inconsistent with the results of a study by Salavatiha et al., which reported HPV 18 A4 variant to be predominant in Iran, [23]. The HPV 18 A1 variants are prevalent in the Asia-Pacific region, and A3 variants account for a large proportion of variants in Europe, Southern/Central Asia, and America [7]. The HPV-16 A1 variants are globally distributed, while other variants have a specific geographic distribution; for example, the A3/A4 variants are predominant in Eastern Asia, and B1–B4/C1–4 variants are frequent in African countries. However, the participants' demographic information was not available, although traveling and immigration are known as significant factors for the geographic distribution of viral variants.

Some studies have reported that mutations in the HPV 16 LCR of non-A variants were associated with an increase in p97 activity and, consequently, an increase in the expression of E6/E7 oncogenes [21]. For instance, in the HPV 16 LCR, an A7727C change was a distinctive characteristic of the increased activity of p97 in D variants [21]. A combination of changes in A7483C, G7487A, G7519A, C7667T, C7687A, A7727C, C7762T, and C7784T led to an increase in the transcription activity of these variants [15]; all of these changes were observed in the studied HPV-16 LCR. Also, Veress et al. showed that many nucleotide changes responsible for the increased transcription activity in HPV16 D and A4 variant genes were related to the LCR 3′ half [13].

Moreover, Lace et al. showed that nucleotide changes in A7483C, G7487A, G7519A, A7505G, C7667T, C7687A, A7727C, T7741G, C7762T, and C7784T affected the ori function of HPV, leading to an increase in ori-dependent transcription and replication activities, increased activities of promoters (by 2–3.5 folds), and consequently, the increased expression of E6 and E7 oncogenes [24]. Among mutations described here, A7483C, G7487A, G7519A, C7667T, C7687A, A7727C G7824A, C7762T, and C7784T mutations were identified in D1 isolates. The findings for HPV 18 indicated no significant association between the variants and invasive cervical cancer [5, 22].

Compared to the reference sequences, mutations within LCR in all subjects had more binding sites for some transcription factors, such as YY1, NF-1, Oct-2.1, TFIID, E2F, and TEF-1. Pande et al. reported that some mutations in the HPV 16 LCR, especially in YY1 binding sites, such as G7519A (the most common mutation in YY1 binding site), A7634C, C7687A, T7712G, C7790T, G7824A, A7837C, T7741, and C7762T, led to increased p97 activity by three to six folds [25]. Among these variants, G7519A, C7687A, and G7824A were identified in our samples. Regarding HPV 18, 64.3% (9/14) of the identified SNPs were in the c-Fos and HMGI binding sites.

Compared to c-Jun family members, C-Fos is an AP-1 transcription factor subunit. Overall, AP-1 is one of the factors, which plays a crucial role in the primary stages of neoplastic changes. All these factors regulate the miR-21 genes in cervical cancer cells, associated with HPV 18 and HPV 16 [26]. Moreover, Wang et al., in their study on cervical samples, showed that the overexpression of HMGA2 led to the progression of cervical intraepithelial neoplasia (CIN) into cervical cancer [27]. It seems that variations in the HPV-18 genome, as well as other high-risk HPV genotypes, can affect the cellular and viral transcription sites, although further research is warranted.

The present study had several limitations. First, this study had a cross-sectional design, with no demographic information available regarding the cancer status of the samples; therefore, more detailed longitudinal studies are suggested at a molecular level. Second, in this study, samples collected in the first national HPV screening program in Iran were used, while the diversity of HPV genotypes in all provinces of Iran was not comprehensively studied. Finally, although LCR is an important regulatory sequence of HPV genome, according to a study by Qu et al., in which HPV sequences were examined in Chinese women, the accuracy of sublineage classification based on the LCR was 94.02% and 100% for HPV 16 and HPV 18, respectively; they suggested that E2 was the best region for HPV 16 sublineage classification [28]. Nevertheless, in the current study, a partial sequence of LCR was investigated. Therefore, it is suggested to classify whole genomes or marked regions, such as E2, for sublineage classification.

## 5. Conclusion

The present study showed that D1 and A3 were the dominant sublineages of HPV 16 and HPV 18, respectively. Also, YY1, TFIID, Oct-2, NF-1, c-Fos, and MBF1 were the most frequent binding sites, influenced by mutations. Regarding the importance of mutations in the LCR of non-A variants in developing higher grades of neoplasia (CIN grade 2/3) or invasive cervical cancer, women infected with these variants should be examined in future longitudinal studies to obtain further information about the oncogenic potential of these dominant variants in Iran.

## Figures and Tables

**Table 1:** All mutations in the studied HPV-16 isolates.

Province	Sublineage/isolate	7358	7359	7371	7382	7384	7387	7393	7431	7433	7434	7439	7448	7483	7487	7519	7667	7687	7727	7762	7782	7783	7784	7797	7824	7832	7835	7837
	HPV-16 Ref	G	G	A	A	T	C	C	A	G	C	T	T	A	G	G	C	C	A	C	C	C	C	G	G	G	A	A
Tehran	D1/STi38	—/A	—/A	—	—	—	—	—	—	—	G	G	—	C	A	A	T	A	C	T	T	—	T	—	—	T	—	—
Tehran	D1/STi27	—	—	—	—	—	—	G	—	—	G	G	—	C	A	A	T	A	C	T	T	A	T	—	—	T	—	—
Khuzestan	D1/TP4450	—	—	—	T	G	—	—	—	—	G	G	—	C	A	A	T	A	C	T	T	—	T	—	—	T	—	—
Kerman	D1/TP542	—	—	—	—	—	—	—	G	—	G	G	—	C	A	A	T	A	C	T	T	—	T	—	—	T	—	—
Ardabil	D1/TP128	—	—	—	—	—	—	—	—	—	G	G	—	C	A	A	T	A	C	T	T	—	T	—	—	T	—	—
Ardabil	D1/TP2325	—	—	—	—	—	—	—	—	—	G	G	—	C	A	A	T	A	C	T	T	—	T	—	—	T	—	—
Ardabil	D1/TP3992	—	—	—	—	—	—	—	—	—	G	G	—	C	A	A	T	A	C	T	T	—	T	—	—	T	—	—
Semnan	D1/TP3144	—	—	—	—	—	—	—	—	—	G	—	—	C	A	A	T	A	C	T	—	—	T	—	A	T	—	—
Ardabil	D1/TP4088	—	—	—	—	—	—	—	—	—	G	—	—	C	A	A	T	A	C	T	—	—	T	—	A	T	—	—
Hormozgan	D1/TP4938	—	—	—	—	—	—	—	—	—	G	—	—	C	A	A	T	A	C	T	—	—	T	—	A	T	—	—
Tehran	D1/STi26	—	—	—	—	—	—	—	—	—	G	—	—	C	A	A	T	A	C	T	—	—	T	—	—	T	—	—
Tehran	D1/STi13	—	—	—	—	—	—	—	—	—	G	G	—	C	A	A	T	A	C	T	T	—	T	—	—	T	—	—
Tehran	D1/STi20	—	—	—	—	—	—	—	—	—	G	G	—	C	A	A	T	A	C	T	T	—	T	—	—	T	—	—
Tehran	D1/STi21	—	—	—	—	—	—	—	—	—	G	G	—	C	A	A	T	A	C	T	T	—	T	—	—	T	—	—
Tehran	D1/STi28	—	—	—	—	—	—	—	—	—	G	G	—	C	A	A	T	A	C	T	T	—	T	—	—	T	—	—
Tehran	D1/STi65	—	—	—	—	—	—	—	—	—	G	G	—	C	A	A	T	A	C	T	T	—	T	—	—	T	—	—
Tehran	D1/STi101	—	—	—	—	—	—	—	—	—	G	G	—	C	A	A	T	A	C	T	T	—	T	—	—	T	—	—
Tehran	D1/STi106	—	—	—	—	—	—	—	—	—	G	G	—	C	A	A	T	A	C	T	T	—	T	—	—	T	—	—
Tehran	D1/STi110	—	—	—	—	—	—	—	—	—	G	G	—	C	A	A	T	A	C	T	T	—	T	—	—	T	—	—
Yazd	D1/TP5246	—	—	—	—	—	—	—	—	—	G	G	—	C	A	A	T	A	C	T	T	—	T	—	—	T	—	—
Khuzestan	D1/TP5304	—	—	—	—	—	—	—	—	—	G	G	—	C	A	A	T	A	C	T	T	—	T	—	—	T	—	—
Gilan	D1/TP2198	—	—	—	—	—	—	—	—	—	G	G	—	C	A	A	T	A	C	T	T	—	T	—	—	T	—	—
Isfahan	D1/TP3888	—	—	—	—	—	—	—	—	—	G	G	—	C	A	A	T	A	C	T	T	—	T	—	—	T	—	—
Khuzestan	D1/TP4494	—	—	—	—	—	—	—	—	—	G	G	—	C	A	A	T	A	C	T	T	—	T	—	—	T	—	—
Yazd	D1/TP5124	—	—	—	—	—	—	—	—	—	G	G	—	C	A	A	T	A	C	T	T	—	T	—	—	T	—	—
Kerman	D1/TP4822	—	—	—	—	—	—	—	—	—	G	G	—	C	A	A	T	A	C	T	T	—	T	—	—	T	—	—
Gilan	D1/TP3376	—	—	—	—	—	—	—	—	—	G	G	—	C	A	A	T	A	C	T	T	—	T	—	—	T	—	—
Khuzestan	D1/TP2790	—	—	—	—	—	—	—	—	—	G	G	—	C	A	A	T	A	C	T	T	—	T	—	—	T	—	—
Gilan	D1/TP2219	—	—	—	—	—	—	—	—	—	G	G	—	C	A	A	T	A	C	T	T	—	T	—	—	T	—	—
Gilan	D1/TP2249	—	—	—	—	—	—	—	—	—	G	G	—	C	A	A	T	A	C	T	T	—	T	—	—	T	—	—
East Azerbaijan	D1/TP2067	—	—	—	—	—	—	—	—	—	G	G	—	C	A	A	T	A	C	T	T	—	T	A	—	T	—	—
Ardabil	A4/TP130	—	—	—	—	—	—	—	—	—	G	—	—	—	—	A	—	—	—	—	—	—	—	—	—	—	—	—
Khuzestan	A4/TP3700	—	—	—	—	—	T	—	—	—	G	—	—	—	—	A	—	—	—	—	—	—	—	—	—	—	—	—
Kerman	A4/TP4904	—	—	—	—	—	—	—	—	—	G	—	—	—	—	A	—	—	—	—	—	—	—	—	—	—	—	—
Tehran	A4/STi62	—	—	—	—	—	—	—	—	—	G	—	—	—	—	A	—	—	—	—	—	—	—	—	—	—	—	—
Yazd	A4/TP5210	—	—	—	—	—	—	—	—	—	G	—	C	—	—	A	—	—	—	—	—	—	—	—	—	—	—	—
Kurdistan	A1/TP228	—	—	—	—	—	—	—	—	—	G	—	—	—	—	—	—	—	—	—	—	—	—	—	—	—	—	—
Ardabil	C1/TP86	—	—	C	—	—	—	—	—	A	G	—	—	C	A	A	T	A	—	T	—	—	T	—	A	T	C	G

**Table 2:** All mutations in the studied HPV-18 isolates.

Lineage	Sub-lineage	Province	Isolate	7486	7527	7529	7530	7563	7567	7577	7592	7670	7857	7897	7898	7943	7961
	HPV-18 ref			C	A	C	T	G	A	C	T	A	C	T	A	A	T
A	A3	Tehran	STi7	T	—	A	—	—	C	—	C	T	—	G	—	—	C
		Tehran	STi26	T	—	A	-	A	C	—	C	T	—	—	—	—	C
		Tehran	STi33	T	C	A	G	A	C	—	C	T	—	—	—	T	C
		Tehran	STi38	T	—	A	—	—	C	—	C	T	—	—	—	—	C
		Tehran	STi55	T	—	A	G	—	C	—	C	T	—	—	—	—	C
		Tehran	STi101	T	—	A	—	A	C	—	C	T	—	—	—	—	C
		Tehran	STi94	T	—	A	—	A	C	—	C	T	—	—	—	—	C
		Gilan	TP2204	T	—	A	—	A	C	—	C	T	—	—	—	—	C
		Semnan	TP3272	T	—	A	G	—	C	—	C	T	T	—	—	—	C
		Hormozgan	TP4244	T	—	A	—	A	C	—	C	T	—	—	—	—	C
		Hormozgan	TP4936	T	—	A	—	A	C	—	C	T	—	—	—	—	C
		Khuzestan	TP4986	T	—	A	—	A	C	A	C	T	—	—	—	—	C
		Khuzestan	TP5304	T	—	A	—	A	C	—	C	T	—	—	—	—	C
	A4	Tehran	STi119	—	—	A	—	—	C	—	C	T	—	—	G	—	C

## Data Availability

The data that supported the findings of the current study are available in GenBank and the cited references.

## Supplementary Materials

Supplementary Materials
*Supplementary Table 1a*. All the transcription binding sites and factors in the HPV-18 LCR. *Supplementary Table 1b*. All the transcription binding sites and factors in the HPV-16 LCR.
