# Supplementary material for: Genetic Diversity of HPV 16 and HPV 18 Based on Partial Long Control Region in Iranian Women
Source: Can J Infect Dis Med Microbiol. 2022 Jan 25;2022:4759871. doi: 10.1155/2022/4759871 (PMC8808245; doi:10.1155/2022/4759871)
Supplement: Supplementary Materials — Supplementary Table 1a. All the transcription binding sites and factors in the HPV-18 LCR. Supplementary Table 1b. All the transcription binding sites and factors in the HPV-16 LCR. [file 4759871.f1.docx]

supplementary Table 1a. All the transcription binding sites and factors in the HPV-18 LCR.

| Isolates | 7485 | 7495 | 7525 | 7535 | 7545 | 7565 | 7575 | 7585 | 7595 | 7615 | 7625 | 7645 | 7685 | 7695 | 7705 | 7715 | 7725 | 7735 | 7755 | 7765 | 7795 | 7805 | 7815 | 7825 | 7835 | 7845 | 7855 | 7865 | 7885 | 7895 | 7905 | 7915 | 7925 | 7935 |
| --- | --- | --- | --- | --- | --- | --- | --- | --- | --- | --- | --- | --- | --- | --- | --- | --- | --- | --- | --- | --- | --- | --- | --- | --- | --- | --- | --- | --- | --- | --- | --- | --- | --- | --- |
| Ref | \| 0 \|  \|  \|  \| \| --- \| --- \| --- \| --- \| | \| 1 \| 2 \| 7 \|  \| \| --- \| --- \| --- \| --- \| | \| 1 \|  \|  \|  \| \| --- \| --- \| --- \| --- \| | \| 2 \| 7 \|  \|  \| \| --- \| --- \| --- \| --- \| | \| 2 \|  \|  \|  \| \| --- \| --- \| --- \| --- \| | \| 2 \| 7 \|  \|  \| \| --- \| --- \| --- \| --- \| | \| 0 \|  \|  \|  \| \| --- \| --- \| --- \| --- \| | \| 0 \| 13 \|  \|  \| \| --- \| --- \| --- \| --- \| | \| 3 \| 4 \| 5 \|  \| \| --- \| --- \| --- \| --- \| | \| 2 \| 6 \| 7 \| 8 \| \| --- \| --- \| --- \| --- \| | \| 1 \|  \|  \|  \| \| --- \| --- \| --- \| --- \| | \| 9 \|  \|  \|  \| \| --- \| --- \| --- \| --- \| | \| 2 \| 7 \|  \|  \| \| --- \| --- \| --- \| --- \| | \| 2 \| 7 \| 12 \|  \| \| --- \| --- \| --- \| --- \| | \| 2 \| 7 \|  \|  \| \| --- \| --- \| --- \| --- \| | \| 1 \| 2 \| 7 \|  \| \| --- \| --- \| --- \| --- \| | \| 9 \|  \|  \|  \| \| --- \| --- \| --- \| --- \| | \| 0 \|  \|  \|  \| \| --- \| --- \| --- \| --- \| | \| 2 \|  \|  \|  \| \| --- \| --- \| --- \| --- \| | \| 11 \|  \|  \|  \| \| --- \| --- \| --- \| --- \| | \| 3 \| 4 \| 6 \| 8 \| \| --- \| --- \| --- \| --- \| | \| 9 \|  \|  \|  \| \| --- \| --- \| --- \| --- \| | \| 2 \|  \|  \|  \| \| --- \| --- \| --- \| --- \| | \| 9 \|  \|  \|  \| \| --- \| --- \| --- \| --- \| | \| 2 \|  \|  \|  \| \| --- \| --- \| --- \| --- \| | \| 11 \|  \|  \|  \| \| --- \| --- \| --- \| --- \| | \| 1 \| 2 \| 7 \|  \| \| --- \| --- \| --- \| --- \| | \| 2 \| 7 \|  \|  \| \| --- \| --- \| --- \| --- \| | \| 3 \| 4 \|  \|  \| \| --- \| --- \| --- \| --- \| | \| 2 \| 7 \|  \|  \| \| --- \| --- \| --- \| --- \| | \| 1 \| 2 \| 7 \|  \| \| --- \| --- \| --- \| --- \| | \| 10 \|  \|  \|  \| \| --- \| --- \| --- \| --- \| | \| 1 \| 2 \| 7 \|  \| \| --- \| --- \| --- \| --- \| | \| 2 \| 3 \| 4 \| 7 \| \| --- \| --- \| --- \| --- \| |
| STi7 | \| 0 \| 2 \| 12 \|  \| \| --- \| --- \| --- \| --- \| | \| 1 \| 2 \| 7 \|  \| \| --- \| --- \| --- \| --- \| | \| 1 \|  \|  \|  \| \| --- \| --- \| --- \| --- \| | \| 2 \| 7 \|  \|  \| \| --- \| --- \| --- \| --- \| | \| 2 \|  \|  \|  \| \| --- \| --- \| --- \| --- \| | \| 2 \| 7 \|  \|  \| \| --- \| --- \| --- \| --- \| | \| 0 \|  \|  \|  \| \| --- \| --- \| --- \| --- \| | \| 0 \| 13 \|  \|  \| \| --- \| --- \| --- \| --- \| | \| 5 \|  \|  \|  \| \| --- \| --- \| --- \| --- \| | \| 2 \| 6 \| 7 \| 8 \| \| --- \| --- \| --- \| --- \| | \| 1 \|  \|  \|  \| \| --- \| --- \| --- \| --- \| | \| 9 \|  \|  \|  \| \| --- \| --- \| --- \| --- \| | \| 2 \| 7 \|  \|  \| \| --- \| --- \| --- \| --- \| | \| 2 \| 7 \| 12 \|  \| \| --- \| --- \| --- \| --- \| | \| 2 \| 7 \|  \|  \| \| --- \| --- \| --- \| --- \| | \| 1 \| 2 \| 7 \|  \| \| --- \| --- \| --- \| --- \| | \| 9 \|  \|  \|  \| \| --- \| --- \| --- \| --- \| | \| 0 \|  \|  \|  \| \| --- \| --- \| --- \| --- \| | \| 2 \|  \|  \|  \| \| --- \| --- \| --- \| --- \| | \| 11 \|  \|  \|  \| \| --- \| --- \| --- \| --- \| | \| 3 \| 4 \| 6 \| 8 \| \| --- \| --- \| --- \| --- \| | \| 9 \|  \|  \|  \| \| --- \| --- \| --- \| --- \| | \| 2 \|  \|  \|  \| \| --- \| --- \| --- \| --- \| | \| 9 \|  \|  \|  \| \| --- \| --- \| --- \| --- \| | \| 2 \|  \|  \|  \| \| --- \| --- \| --- \| --- \| | \| 11 \|  \|  \|  \| \| --- \| --- \| --- \| --- \| | \| 1 \| 2 \| 7 \|  \| \| --- \| --- \| --- \| --- \| | \| 2 \| 7 \|  \|  \| \| --- \| --- \| --- \| --- \| | \| 3 \| 4 \|  \|  \| \| --- \| --- \| --- \| --- \| | \| 2 \| 7 \|  \|  \| \| --- \| --- \| --- \| --- \| | \| 1 \| 2 \| 7 \|  \| \| --- \| --- \| --- \| --- \| | \| 10 \|  \|  \|  \| \| --- \| --- \| --- \| --- \| | \| 1 \| 2 \| 7 \|  \| \| --- \| --- \| --- \| --- \| | \| 2 \| 3 \| 4 \| 7 \| \| --- \| --- \| --- \| --- \| |
| STi26 | \| 0 \| 2 \| 12 \|  \| \| --- \| --- \| --- \| --- \| | \| 1 \| 2 \| 7 \|  \| \| --- \| --- \| --- \| --- \| | \| 1 \|  \|  \|  \| \| --- \| --- \| --- \| --- \| | \| 2 \| 7 \|  \|  \| \| --- \| --- \| --- \| --- \| | \| 2 \|  \|  \|  \| \| --- \| --- \| --- \| --- \| | \| 2 \| 7 \|  \|  \| \| --- \| --- \| --- \| --- \| | \| 0 \|  \|  \|  \| \| --- \| --- \| --- \| --- \| | \| 0 \| 13 \|  \|  \| \| --- \| --- \| --- \| --- \| | \| 5 \|  \|  \|  \| \| --- \| --- \| --- \| --- \| | \| 2 \| 6 \| 7 \| 8 \| \| --- \| --- \| --- \| --- \| | \| 1 \|  \|  \|  \| \| --- \| --- \| --- \| --- \| | \| 9 \|  \|  \|  \| \| --- \| --- \| --- \| --- \| | \| 2 \| 7 \|  \|  \| \| --- \| --- \| --- \| --- \| | \| 2 \| 7 \| 12 \|  \| \| --- \| --- \| --- \| --- \| | \| 2 \| 7 \|  \|  \| \| --- \| --- \| --- \| --- \| | \| 1 \| 2 \| 7 \|  \| \| --- \| --- \| --- \| --- \| | \| 9 \|  \|  \|  \| \| --- \| --- \| --- \| --- \| | \| 0 \|  \|  \|  \| \| --- \| --- \| --- \| --- \| | \| 2 \|  \|  \|  \| \| --- \| --- \| --- \| --- \| | \| 11 \|  \|  \|  \| \| --- \| --- \| --- \| --- \| | \| 3 \| 4 \| 6 \| 8 \| \| --- \| --- \| --- \| --- \| | \| 9 \|  \|  \|  \| \| --- \| --- \| --- \| --- \| | \| 2 \|  \|  \|  \| \| --- \| --- \| --- \| --- \| | \| 9 \|  \|  \|  \| \| --- \| --- \| --- \| --- \| | \| 2 \|  \|  \|  \| \| --- \| --- \| --- \| --- \| | \| 11 \|  \|  \|  \| \| --- \| --- \| --- \| --- \| | \| 1 \| 2 \| 7 \|  \| \| --- \| --- \| --- \| --- \| | \| 2 \| 7 \|  \|  \| \| --- \| --- \| --- \| --- \| | \| 3 \| 4 \|  \|  \| \| --- \| --- \| --- \| --- \| | \| 2 \| 7 \|  \|  \| \| --- \| --- \| --- \| --- \| | \| 1 \| 2 \| 7 \|  \| \| --- \| --- \| --- \| --- \| | \| 10 \|  \|  \|  \| \| --- \| --- \| --- \| --- \| | \| 1 \| 2 \| 7 \|  \| \| --- \| --- \| --- \| --- \| | \| 2 \| 3 \| 4 \| 7 \| \| --- \| --- \| --- \| --- \| |
| STi33 | \| 0 \| 2 \| 12 \|  \| \| --- \| --- \| --- \| --- \| | \| 1 \| 2 \| 7 \|  \| \| --- \| --- \| --- \| --- \| | \|  \|  \|  \|  \| \| --- \| --- \| --- \| --- \| | \| 2 \| 7 \|  \|  \| \| --- \| --- \| --- \| --- \| | \| 2 \|  \|  \|  \| \| --- \| --- \| --- \| --- \| | \| 2 \| 7 \|  \|  \| \| --- \| --- \| --- \| --- \| | \| 0 \|  \|  \|  \| \| --- \| --- \| --- \| --- \| | \| 0 \| 13 \|  \|  \| \| --- \| --- \| --- \| --- \| | \| 5 \|  \|  \|  \| \| --- \| --- \| --- \| --- \| | \| 2 \| 6 \| 7 \| 8 \| \| --- \| --- \| --- \| --- \| | \| 1 \|  \|  \|  \| \| --- \| --- \| --- \| --- \| | \| 9 \|  \|  \|  \| \| --- \| --- \| --- \| --- \| | \| 2 \| 7 \|  \|  \| \| --- \| --- \| --- \| --- \| | \| 2 \| 7 \| 12 \|  \| \| --- \| --- \| --- \| --- \| | \| 2 \| 7 \|  \|  \| \| --- \| --- \| --- \| --- \| | \| 1 \| 2 \| 7 \|  \| \| --- \| --- \| --- \| --- \| | \| 9 \|  \|  \|  \| \| --- \| --- \| --- \| --- \| | \| 0 \|  \|  \|  \| \| --- \| --- \| --- \| --- \| | \| 2 \|  \|  \|  \| \| --- \| --- \| --- \| --- \| | \| 11 \|  \|  \|  \| \| --- \| --- \| --- \| --- \| | \| 3 \| 4 \| 6 \| 8 \| \| --- \| --- \| --- \| --- \| | \| 9 \|  \|  \|  \| \| --- \| --- \| --- \| --- \| | \| 2 \|  \|  \|  \| \| --- \| --- \| --- \| --- \| | \| 9 \|  \|  \|  \| \| --- \| --- \| --- \| --- \| | \| 2 \|  \|  \|  \| \| --- \| --- \| --- \| --- \| | \| 11 \|  \|  \|  \| \| --- \| --- \| --- \| --- \| | \| 1 \| 2 \| 7 \|  \| \| --- \| --- \| --- \| --- \| | \| 2 \| 7 \|  \|  \| \| --- \| --- \| --- \| --- \| | \| 3 \| 4 \|  \|  \| \| --- \| --- \| --- \| --- \| | \| 2 \| 7 \|  \|  \| \| --- \| --- \| --- \| --- \| | \| 1 \| 2 \| 7 \|  \| \| --- \| --- \| --- \| --- \| | \| 10 \|  \|  \|  \| \| --- \| --- \| --- \| --- \| | \| 1 \| 2 \| 7 \|  \| \| --- \| --- \| --- \| --- \| | \| 2 \| 3 \| 4 \| 7 \| \| --- \| --- \| --- \| --- \| |
| STi38 | \| 0 \| 2 \| 12 \|  \| \| --- \| --- \| --- \| --- \| | \| 1 \| 2 \| 7 \|  \| \| --- \| --- \| --- \| --- \| | \| 1 \|  \|  \|  \| \| --- \| --- \| --- \| --- \| | \| 2 \| 7 \|  \|  \| \| --- \| --- \| --- \| --- \| | \| 2 \|  \|  \|  \| \| --- \| --- \| --- \| --- \| | \| 2 \| 7 \|  \|  \| \| --- \| --- \| --- \| --- \| | \| 0 \|  \|  \|  \| \| --- \| --- \| --- \| --- \| | \| 0 \| 13 \|  \|  \| \| --- \| --- \| --- \| --- \| | \| 5 \|  \|  \|  \| \| --- \| --- \| --- \| --- \| | \| 2 \| 6 \| 7 \| 8 \| \| --- \| --- \| --- \| --- \| | \| 1 \|  \|  \|  \| \| --- \| --- \| --- \| --- \| | \| 9 \|  \|  \|  \| \| --- \| --- \| --- \| --- \| | \| 2 \| 7 \|  \|  \| \| --- \| --- \| --- \| --- \| | \| 2 \| 7 \| 12 \|  \| \| --- \| --- \| --- \| --- \| | \| 2 \| 7 \|  \|  \| \| --- \| --- \| --- \| --- \| | \| 1 \| 2 \| 7 \|  \| \| --- \| --- \| --- \| --- \| | \| 9 \|  \|  \|  \| \| --- \| --- \| --- \| --- \| | \| 0 \|  \|  \|  \| \| --- \| --- \| --- \| --- \| | \| 2 \|  \|  \|  \| \| --- \| --- \| --- \| --- \| | \| 11 \|  \|  \|  \| \| --- \| --- \| --- \| --- \| | \| 3 \| 4 \| 6 \| 8 \| \| --- \| --- \| --- \| --- \| | \| 9 \|  \|  \|  \| \| --- \| --- \| --- \| --- \| | \| 2 \|  \|  \|  \| \| --- \| --- \| --- \| --- \| | \| 9 \|  \|  \|  \| \| --- \| --- \| --- \| --- \| | \| 2 \|  \|  \|  \| \| --- \| --- \| --- \| --- \| | \| 11 \|  \|  \|  \| \| --- \| --- \| --- \| --- \| | \| 1 \| 2 \| 7 \|  \| \| --- \| --- \| --- \| --- \| | \| 2 \| 7 \|  \|  \| \| --- \| --- \| --- \| --- \| | \| 3 \| 4 \|  \|  \| \| --- \| --- \| --- \| --- \| | \| 2 \| 7 \|  \|  \| \| --- \| --- \| --- \| --- \| | \| 1 \| 2 \| 7 \|  \| \| --- \| --- \| --- \| --- \| | \| 10 \|  \|  \|  \| \| --- \| --- \| --- \| --- \| | \| 1 \| 2 \| 7 \|  \| \| --- \| --- \| --- \| --- \| | \| 2 \| 3 \| 4 \| 7 \| \| --- \| --- \| --- \| --- \| |
| STi55 | \| 0 \| 2 \| 12 \|  \| \| --- \| --- \| --- \| --- \| | \| 1 \| 2 \| 7 \|  \| \| --- \| --- \| --- \| --- \| | \| 1 \|  \|  \|  \| \| --- \| --- \| --- \| --- \| | \| 2 \| 7 \|  \|  \| \| --- \| --- \| --- \| --- \| | \| 2 \|  \|  \|  \| \| --- \| --- \| --- \| --- \| | \| 2 \| 7 \|  \|  \| \| --- \| --- \| --- \| --- \| | \| 0 \|  \|  \|  \| \| --- \| --- \| --- \| --- \| | \| 0 \| 13 \|  \|  \| \| --- \| --- \| --- \| --- \| | \| 5 \|  \|  \|  \| \| --- \| --- \| --- \| --- \| | \| 2 \| 6 \| 7 \| 8 \| \| --- \| --- \| --- \| --- \| | \| 1 \|  \|  \|  \| \| --- \| --- \| --- \| --- \| | \| 9 \|  \|  \|  \| \| --- \| --- \| --- \| --- \| | \| 2 \| 7 \|  \|  \| \| --- \| --- \| --- \| --- \| | \| 2 \| 7 \| 12 \|  \| \| --- \| --- \| --- \| --- \| | \| 2 \| 7 \|  \|  \| \| --- \| --- \| --- \| --- \| | \| 1 \| 2 \| 7 \|  \| \| --- \| --- \| --- \| --- \| | \| 9 \|  \|  \|  \| \| --- \| --- \| --- \| --- \| | \| 0 \|  \|  \|  \| \| --- \| --- \| --- \| --- \| | \| 2 \|  \|  \|  \| \| --- \| --- \| --- \| --- \| | \| 11 \|  \|  \|  \| \| --- \| --- \| --- \| --- \| | \| 3 \| 4 \| 6 \| 8 \| \| --- \| --- \| --- \| --- \| | \| 9 \|  \|  \|  \| \| --- \| --- \| --- \| --- \| | \| 2 \|  \|  \|  \| \| --- \| --- \| --- \| --- \| | \| 9 \|  \|  \|  \| \| --- \| --- \| --- \| --- \| | \| 2 \|  \|  \|  \| \| --- \| --- \| --- \| --- \| | \| 11 \|  \|  \|  \| \| --- \| --- \| --- \| --- \| | \| 1 \| 2 \| 7 \|  \| \| --- \| --- \| --- \| --- \| | \| 2 \| 7 \|  \|  \| \| --- \| --- \| --- \| --- \| | \| 3 \| 4 \|  \|  \| \| --- \| --- \| --- \| --- \| | \| 2 \| 7 \|  \|  \| \| --- \| --- \| --- \| --- \| | \| 1 \| 2 \| 7 \|  \| \| --- \| --- \| --- \| --- \| | \| 10 \|  \|  \|  \| \| --- \| --- \| --- \| --- \| | \| 1 \| 2 \| 7 \|  \| \| --- \| --- \| --- \| --- \| | \| 2 \| 3 \| 4 \| 7 \| \| --- \| --- \| --- \| --- \| |
| STi101 | \| 0 \| 2 \| 12 \|  \| \| --- \| --- \| --- \| --- \| | \| 1 \| 2 \| 7 \|  \| \| --- \| --- \| --- \| --- \| | \| 1 \|  \|  \|  \| \| --- \| --- \| --- \| --- \| | \| 2 \| 7 \|  \|  \| \| --- \| --- \| --- \| --- \| | \| 2 \|  \|  \|  \| \| --- \| --- \| --- \| --- \| | \| 2 \| 7 \|  \|  \| \| --- \| --- \| --- \| --- \| | \| 0 \|  \|  \|  \| \| --- \| --- \| --- \| --- \| | \| 0 \| 13 \|  \|  \| \| --- \| --- \| --- \| --- \| | \| 5 \|  \|  \|  \| \| --- \| --- \| --- \| --- \| | \| 2 \| 6 \| 7 \| 8 \| \| --- \| --- \| --- \| --- \| | \| 1 \|  \|  \|  \| \| --- \| --- \| --- \| --- \| | \| 9 \|  \|  \|  \| \| --- \| --- \| --- \| --- \| | \| 2 \| 7 \|  \|  \| \| --- \| --- \| --- \| --- \| | \| 2 \| 7 \| 12 \|  \| \| --- \| --- \| --- \| --- \| | \| 2 \| 7 \|  \|  \| \| --- \| --- \| --- \| --- \| | \| 1 \| 2 \| 7 \|  \| \| --- \| --- \| --- \| --- \| | \| 9 \|  \|  \|  \| \| --- \| --- \| --- \| --- \| | \| 0 \|  \|  \|  \| \| --- \| --- \| --- \| --- \| | \| 2 \|  \|  \|  \| \| --- \| --- \| --- \| --- \| | \| 11 \|  \|  \|  \| \| --- \| --- \| --- \| --- \| | \| 3 \| 4 \| 6 \| 8 \| \| --- \| --- \| --- \| --- \| | \| 9 \|  \|  \|  \| \| --- \| --- \| --- \| --- \| | \| 2 \|  \|  \|  \| \| --- \| --- \| --- \| --- \| | \| 9 \|  \|  \|  \| \| --- \| --- \| --- \| --- \| | \| 2 \|  \|  \|  \| \| --- \| --- \| --- \| --- \| | \| 11 \|  \|  \|  \| \| --- \| --- \| --- \| --- \| | \| 1 \| 2 \| 7 \|  \| \| --- \| --- \| --- \| --- \| | \| 2 \| 7 \|  \|  \| \| --- \| --- \| --- \| --- \| | \| 3 \| 4 \|  \|  \| \| --- \| --- \| --- \| --- \| | \| 2 \| 7 \|  \|  \| \| --- \| --- \| --- \| --- \| | \| 1 \| 2 \| 7 \|  \| \| --- \| --- \| --- \| --- \| | \| 10 \|  \|  \|  \| \| --- \| --- \| --- \| --- \| | \| 1 \| 2 \| 7 \|  \| \| --- \| --- \| --- \| --- \| | \| 2 \| 3 \| 4 \| 7 \| \| --- \| --- \| --- \| --- \| |
| STi119 | \| 0 \|  \|  \|  \| \| --- \| --- \| --- \| --- \| | \| 1 \| 2 \| 7 \|  \| \| --- \| --- \| --- \| --- \| | \| 1 \|  \|  \|  \| \| --- \| --- \| --- \| --- \| | \| 2 \| 7 \|  \|  \| \| --- \| --- \| --- \| --- \| | \| 2 \|  \|  \|  \| \| --- \| --- \| --- \| --- \| | \| 2 \| 7 \|  \|  \| \| --- \| --- \| --- \| --- \| | \| 0 \|  \|  \|  \| \| --- \| --- \| --- \| --- \| | \| 0 \| 13 \|  \|  \| \| --- \| --- \| --- \| --- \| | \| 5 \|  \|  \|  \| \| --- \| --- \| --- \| --- \| | \| 2 \| 6 \| 7 \| 8 \| \| --- \| --- \| --- \| --- \| | \| 1 \|  \|  \|  \| \| --- \| --- \| --- \| --- \| | \| 9 \|  \|  \|  \| \| --- \| --- \| --- \| --- \| | \| 2 \| 7 \|  \|  \| \| --- \| --- \| --- \| --- \| | \| 2 \| 7 \| 12 \|  \| \| --- \| --- \| --- \| --- \| | \| 2 \| 7 \|  \|  \| \| --- \| --- \| --- \| --- \| | \| 1 \| 2 \| 7 \|  \| \| --- \| --- \| --- \| --- \| | \| 9 \|  \|  \|  \| \| --- \| --- \| --- \| --- \| | \| 0 \|  \|  \|  \| \| --- \| --- \| --- \| --- \| | \| 2 \|  \|  \|  \| \| --- \| --- \| --- \| --- \| | \| 11 \|  \|  \|  \| \| --- \| --- \| --- \| --- \| | \| 3 \| 4 \| 6 \| 8 \| \| --- \| --- \| --- \| --- \| | \| 9 \|  \|  \|  \| \| --- \| --- \| --- \| --- \| | \| 2 \|  \|  \|  \| \| --- \| --- \| --- \| --- \| | \| 9 \|  \|  \|  \| \| --- \| --- \| --- \| --- \| | \| 2 \|  \|  \|  \| \| --- \| --- \| --- \| --- \| | \| 11 \|  \|  \|  \| \| --- \| --- \| --- \| --- \| | \| 1 \| 2 \| 7 \|  \| \| --- \| --- \| --- \| --- \| | \| 2 \| 7 \|  \|  \| \| --- \| --- \| --- \| --- \| | \| 3 \| 4 \|  \|  \| \| --- \| --- \| --- \| --- \| | \| 2 \| 7 \|  \|  \| \| --- \| --- \| --- \| --- \| | \| 1 \| 2 \| 7 \| 8 \| \| --- \| --- \| --- \| --- \| | \| 10 \|  \|  \|  \| \| --- \| --- \| --- \| --- \| | \| 1 \| 2 \| 7 \|  \| \| --- \| --- \| --- \| --- \| | \| 2 \| 3 \| 4 \| 7 \| \| --- \| --- \| --- \| --- \| |
| STi94 | \| 0 \| 2 \| 12 \|  \| \| --- \| --- \| --- \| --- \| | \| 1 \| 2 \| 7 \|  \| \| --- \| --- \| --- \| --- \| | \| 1 \|  \|  \|  \| \| --- \| --- \| --- \| --- \| | \| 2 \| 7 \|  \|  \| \| --- \| --- \| --- \| --- \| | \| 2 \|  \|  \|  \| \| --- \| --- \| --- \| --- \| | \| 2 \| 7 \|  \|  \| \| --- \| --- \| --- \| --- \| | \| 0 \|  \|  \|  \| \| --- \| --- \| --- \| --- \| | \| 0 \| 13 \|  \|  \| \| --- \| --- \| --- \| --- \| | \| 5 \|  \|  \|  \| \| --- \| --- \| --- \| --- \| | \| 2 \| 6 \| 7 \| 8 \| \| --- \| --- \| --- \| --- \| | \| 1 \|  \|  \|  \| \| --- \| --- \| --- \| --- \| | \| 9 \|  \|  \|  \| \| --- \| --- \| --- \| --- \| | \| 2 \| 7 \|  \|  \| \| --- \| --- \| --- \| --- \| | \| 2 \| 7 \| 12 \|  \| \| --- \| --- \| --- \| --- \| | \| 2 \| 7 \|  \|  \| \| --- \| --- \| --- \| --- \| | \| 1 \| 2 \| 7 \|  \| \| --- \| --- \| --- \| --- \| | \| 9 \|  \|  \|  \| \| --- \| --- \| --- \| --- \| | \| 0 \|  \|  \|  \| \| --- \| --- \| --- \| --- \| | \| 2 \|  \|  \|  \| \| --- \| --- \| --- \| --- \| | \| 11 \|  \|  \|  \| \| --- \| --- \| --- \| --- \| | \| 3 \| 4 \| 6 \| 8 \| \| --- \| --- \| --- \| --- \| | \| 9 \|  \|  \|  \| \| --- \| --- \| --- \| --- \| | \| 2 \|  \|  \|  \| \| --- \| --- \| --- \| --- \| | \| 9 \|  \|  \|  \| \| --- \| --- \| --- \| --- \| | \| 2 \|  \|  \|  \| \| --- \| --- \| --- \| --- \| | \| 11 \|  \|  \|  \| \| --- \| --- \| --- \| --- \| | \| 1 \| 2 \| 7 \|  \| \| --- \| --- \| --- \| --- \| | \| 2 \| 7 \|  \|  \| \| --- \| --- \| --- \| --- \| | \| 3 \| 4 \|  \|  \| \| --- \| --- \| --- \| --- \| | \| 2 \| 7 \|  \|  \| \| --- \| --- \| --- \| --- \| | \| 1 \| 2 \| 7 \|  \| \| --- \| --- \| --- \| --- \| | \| 10 \|  \|  \|  \| \| --- \| --- \| --- \| --- \| | \| 1 \| 2 \| 7 \|  \| \| --- \| --- \| --- \| --- \| | \| 2 \| 3 \| 4 \| 7 \| \| --- \| --- \| --- \| --- \| |
| TP3272 | \| 0 \| 2 \| 12 \|  \| \| --- \| --- \| --- \| --- \| | \| 1 \| 2 \| 7 \|  \| \| --- \| --- \| --- \| --- \| | \| 1 \|  \|  \|  \| \| --- \| --- \| --- \| --- \| | \| 2 \| 7 \|  \|  \| \| --- \| --- \| --- \| --- \| | \| 2 \|  \|  \|  \| \| --- \| --- \| --- \| --- \| | \| 2 \| 7 \|  \|  \| \| --- \| --- \| --- \| --- \| | \| 0 \|  \|  \|  \| \| --- \| --- \| --- \| --- \| | \| 0 \| 13 \|  \|  \| \| --- \| --- \| --- \| --- \| | \| 5 \|  \|  \|  \| \| --- \| --- \| --- \| --- \| | \| 2 \| 6 \| 7 \| 8 \| \| --- \| --- \| --- \| --- \| | \| 1 \|  \|  \|  \| \| --- \| --- \| --- \| --- \| | \| 9 \|  \|  \|  \| \| --- \| --- \| --- \| --- \| | \| 2 \| 7 \|  \|  \| \| --- \| --- \| --- \| --- \| | \| 2 \| 7 \| 12 \|  \| \| --- \| --- \| --- \| --- \| | \| 2 \| 7 \|  \|  \| \| --- \| --- \| --- \| --- \| | \| 1 \| 2 \| 7 \|  \| \| --- \| --- \| --- \| --- \| | \| 9 \|  \|  \|  \| \| --- \| --- \| --- \| --- \| | \| 0 \|  \|  \|  \| \| --- \| --- \| --- \| --- \| | \| 2 \|  \|  \|  \| \| --- \| --- \| --- \| --- \| | \| 11 \|  \|  \|  \| \| --- \| --- \| --- \| --- \| | \| 3 \| 4 \| 6 \| 8 \| \| --- \| --- \| --- \| --- \| | \| 9 \|  \|  \|  \| \| --- \| --- \| --- \| --- \| | \| 2 \|  \|  \|  \| \| --- \| --- \| --- \| --- \| | \| 9 \|  \|  \|  \| \| --- \| --- \| --- \| --- \| | \| 2 \|  \|  \|  \| \| --- \| --- \| --- \| --- \| | \| 11 \|  \|  \|  \| \| --- \| --- \| --- \| --- \| | \| 2 \| 7 \|  \|  \| \| --- \| --- \| --- \| --- \| | \| 2 \| 7 \|  \|  \| \| --- \| --- \| --- \| --- \| | \| 3 \| 4 \|  \|  \| \| --- \| --- \| --- \| --- \| | \| 2 \| 7 \|  \|  \| \| --- \| --- \| --- \| --- \| | \| 1 \| 2 \| 7 \|  \| \| --- \| --- \| --- \| --- \| | \| 10 \|  \|  \|  \| \| --- \| --- \| --- \| --- \| | \| 1 \| 2 \| 7 \|  \| \| --- \| --- \| --- \| --- \| | \| 2 \| 3 \| 4 \| 7 \| \| --- \| --- \| --- \| --- \| |
| TP4244 | \| 0 \| 2 \| 12 \|  \| \| --- \| --- \| --- \| --- \| | \| 1 \| 2 \| 7 \|  \| \| --- \| --- \| --- \| --- \| | \| 1 \|  \|  \|  \| \| --- \| --- \| --- \| --- \| | \| 2 \| 7 \|  \|  \| \| --- \| --- \| --- \| --- \| | \| 2 \|  \|  \|  \| \| --- \| --- \| --- \| --- \| | \| 2 \| 7 \|  \|  \| \| --- \| --- \| --- \| --- \| | \| 0 \|  \|  \|  \| \| --- \| --- \| --- \| --- \| | \| 0 \| 13 \|  \|  \| \| --- \| --- \| --- \| --- \| | \| 5 \|  \|  \|  \| \| --- \| --- \| --- \| --- \| | \| 2 \| 6 \| 7 \| 8 \| \| --- \| --- \| --- \| --- \| | \| 1 \|  \|  \|  \| \| --- \| --- \| --- \| --- \| | \| 9 \|  \|  \|  \| \| --- \| --- \| --- \| --- \| | \| 2 \| 7 \|  \|  \| \| --- \| --- \| --- \| --- \| | \| 2 \| 7 \| 12 \|  \| \| --- \| --- \| --- \| --- \| | \| 2 \| 7 \|  \|  \| \| --- \| --- \| --- \| --- \| | \| 1 \| 2 \| 7 \|  \| \| --- \| --- \| --- \| --- \| | \| 9 \|  \|  \|  \| \| --- \| --- \| --- \| --- \| | \| 0 \|  \|  \|  \| \| --- \| --- \| --- \| --- \| | \| 2 \|  \|  \|  \| \| --- \| --- \| --- \| --- \| | \| 11 \|  \|  \|  \| \| --- \| --- \| --- \| --- \| | \| 3 \| 4 \| 6 \| 8 \| \| --- \| --- \| --- \| --- \| | \| 9 \|  \|  \|  \| \| --- \| --- \| --- \| --- \| | \| 2 \|  \|  \|  \| \| --- \| --- \| --- \| --- \| | \| 9 \|  \|  \|  \| \| --- \| --- \| --- \| --- \| | \| 2 \|  \|  \|  \| \| --- \| --- \| --- \| --- \| | \| 11 \|  \|  \|  \| \| --- \| --- \| --- \| --- \| | \| 1 \| 2 \| 7 \|  \| \| --- \| --- \| --- \| --- \| | \| 2 \| 7 \|  \|  \| \| --- \| --- \| --- \| --- \| | \| 3 \| 4 \|  \|  \| \| --- \| --- \| --- \| --- \| | \| 2 \| 7 \|  \|  \| \| --- \| --- \| --- \| --- \| | \| 1 \| 2 \| 7 \|  \| \| --- \| --- \| --- \| --- \| | \| 10 \|  \|  \|  \| \| --- \| --- \| --- \| --- \| | \| 1 \| 2 \| 7 \|  \| \| --- \| --- \| --- \| --- \| | \| 2 \| 3 \| 4 \| 7 \| \| --- \| --- \| --- \| --- \| |
| TP4936 | \| 0 \| 2 \| 12 \|  \| \| --- \| --- \| --- \| --- \| | \| 1 \| 2 \| 7 \|  \| \| --- \| --- \| --- \| --- \| | \| 1 \|  \|  \|  \| \| --- \| --- \| --- \| --- \| | \| 2 \| 7 \|  \|  \| \| --- \| --- \| --- \| --- \| | \| 2 \|  \|  \|  \| \| --- \| --- \| --- \| --- \| | \| 2 \| 7 \|  \|  \| \| --- \| --- \| --- \| --- \| | \| 0 \|  \|  \|  \| \| --- \| --- \| --- \| --- \| | \| 0 \| 13 \|  \|  \| \| --- \| --- \| --- \| --- \| | \| 5 \|  \|  \|  \| \| --- \| --- \| --- \| --- \| | \| 2 \| 6 \| 7 \| 8 \| \| --- \| --- \| --- \| --- \| | \| 1 \|  \|  \|  \| \| --- \| --- \| --- \| --- \| | \| 9 \|  \|  \|  \| \| --- \| --- \| --- \| --- \| | \| 2 \| 7 \|  \|  \| \| --- \| --- \| --- \| --- \| | \| 2 \| 7 \| 12 \|  \| \| --- \| --- \| --- \| --- \| | \| 2 \| 7 \|  \|  \| \| --- \| --- \| --- \| --- \| | \| 1 \| 2 \| 7 \|  \| \| --- \| --- \| --- \| --- \| | \| 9 \|  \|  \|  \| \| --- \| --- \| --- \| --- \| | \| 0 \|  \|  \|  \| \| --- \| --- \| --- \| --- \| | \| 2 \|  \|  \|  \| \| --- \| --- \| --- \| --- \| | \| 11 \|  \|  \|  \| \| --- \| --- \| --- \| --- \| | \| 3 \| 4 \| 6 \| 8 \| \| --- \| --- \| --- \| --- \| | \| 9 \|  \|  \|  \| \| --- \| --- \| --- \| --- \| | \| 2 \|  \|  \|  \| \| --- \| --- \| --- \| --- \| | \| 9 \|  \|  \|  \| \| --- \| --- \| --- \| --- \| | \| 2 \|  \|  \|  \| \| --- \| --- \| --- \| --- \| | \| 11 \|  \|  \|  \| \| --- \| --- \| --- \| --- \| | \| 1 \| 2 \| 7 \|  \| \| --- \| --- \| --- \| --- \| | \| 2 \| 7 \|  \|  \| \| --- \| --- \| --- \| --- \| | \| 3 \| 4 \|  \|  \| \| --- \| --- \| --- \| --- \| | \| 2 \| 7 \|  \|  \| \| --- \| --- \| --- \| --- \| | \| 1 \| 2 \| 7 \|  \| \| --- \| --- \| --- \| --- \| | \| 10 \|  \|  \|  \| \| --- \| --- \| --- \| --- \| | \| 1 \| 2 \| 7 \|  \| \| --- \| --- \| --- \| --- \| | \| 2 \| 3 \| 4 \| 7 \| \| --- \| --- \| --- \| --- \| |
| TP4986 | \| 0 \| 2 \| 12 \|  \| \| --- \| --- \| --- \| --- \| | \| 1 \| 2 \| 7 \|  \| \| --- \| --- \| --- \| --- \| | \| 1 \|  \|  \|  \| \| --- \| --- \| --- \| --- \| | \| 2 \| 7 \|  \|  \| \| --- \| --- \| --- \| --- \| | \| 2 \|  \|  \|  \| \| --- \| --- \| --- \| --- \| | \| 2 \| 7 \|  \|  \| \| --- \| --- \| --- \| --- \| | \| 0 \|  \|  \|  \| \| --- \| --- \| --- \| --- \| | \| 0 \| 13 \|  \|  \| \| --- \| --- \| --- \| --- \| | \| 5 \|  \|  \|  \| \| --- \| --- \| --- \| --- \| | \| 2 \| 6 \| 7 \| 8 \| \| --- \| --- \| --- \| --- \| | \| 1 \|  \|  \|  \| \| --- \| --- \| --- \| --- \| | \| 9 \|  \|  \|  \| \| --- \| --- \| --- \| --- \| | \| 2 \| 7 \|  \|  \| \| --- \| --- \| --- \| --- \| | \| 2 \| 7 \| 12 \|  \| \| --- \| --- \| --- \| --- \| | \| 2 \| 7 \|  \|  \| \| --- \| --- \| --- \| --- \| | \| 1 \| 2 \| 7 \|  \| \| --- \| --- \| --- \| --- \| | \| 9 \|  \|  \|  \| \| --- \| --- \| --- \| --- \| | \| 0 \|  \|  \|  \| \| --- \| --- \| --- \| --- \| | \| 2 \|  \|  \|  \| \| --- \| --- \| --- \| --- \| | \| 11 \|  \|  \|  \| \| --- \| --- \| --- \| --- \| | \| 3 \| 4 \| 6 \| 8 \| \| --- \| --- \| --- \| --- \| | \| 9 \|  \|  \|  \| \| --- \| --- \| --- \| --- \| | \| 2 \|  \|  \|  \| \| --- \| --- \| --- \| --- \| | \| 9 \|  \|  \|  \| \| --- \| --- \| --- \| --- \| | \| 2 \|  \|  \|  \| \| --- \| --- \| --- \| --- \| | \| 11 \|  \|  \|  \| \| --- \| --- \| --- \| --- \| | \| 1 \| 2 \| 7 \|  \| \| --- \| --- \| --- \| --- \| | \| 2 \| 7 \|  \|  \| \| --- \| --- \| --- \| --- \| | \| 3 \| 4 \|  \|  \| \| --- \| --- \| --- \| --- \| | \| 2 \| 7 \|  \|  \| \| --- \| --- \| --- \| --- \| | \| 1 \| 2 \| 7 \|  \| \| --- \| --- \| --- \| --- \| | \| 10 \|  \|  \|  \| \| --- \| --- \| --- \| --- \| | \| 1 \| 2 \| 7 \|  \| \| --- \| --- \| --- \| --- \| | \| 2 \| 3 \| 4 \| 7 \| \| --- \| --- \| --- \| --- \| |
| TP5304 | \| 0 \| 2 \| 12 \|  \| \| --- \| --- \| --- \| --- \| | \| 1 \| 2 \| 7 \|  \| \| --- \| --- \| --- \| --- \| | \| 1 \|  \|  \|  \| \| --- \| --- \| --- \| --- \| | \| 2 \| 7 \|  \|  \| \| --- \| --- \| --- \| --- \| | \| 2 \|  \|  \|  \| \| --- \| --- \| --- \| --- \| | \| 2 \| 7 \|  \|  \| \| --- \| --- \| --- \| --- \| | \| 0 \|  \|  \|  \| \| --- \| --- \| --- \| --- \| | \| 0 \| 13 \|  \|  \| \| --- \| --- \| --- \| --- \| | \| 5 \|  \|  \|  \| \| --- \| --- \| --- \| --- \| | \| 2 \| 6 \| 7 \| 8 \| \| --- \| --- \| --- \| --- \| | \| 1 \|  \|  \|  \| \| --- \| --- \| --- \| --- \| | \| 9 \|  \|  \|  \| \| --- \| --- \| --- \| --- \| | \| 2 \| 7 \|  \|  \| \| --- \| --- \| --- \| --- \| | \| 2 \| 7 \| 12 \|  \| \| --- \| --- \| --- \| --- \| | \| 2 \| 7 \|  \|  \| \| --- \| --- \| --- \| --- \| | \| 1 \| 2 \| 7 \|  \| \| --- \| --- \| --- \| --- \| | \| 9 \|  \|  \|  \| \| --- \| --- \| --- \| --- \| | \| 0 \|  \|  \|  \| \| --- \| --- \| --- \| --- \| | \| 2 \|  \|  \|  \| \| --- \| --- \| --- \| --- \| | \| 11 \|  \|  \|  \| \| --- \| --- \| --- \| --- \| | \| 3 \| 4 \| 6 \| 8 \| \| --- \| --- \| --- \| --- \| | \| 9 \|  \|  \|  \| \| --- \| --- \| --- \| --- \| | \| 2 \|  \|  \|  \| \| --- \| --- \| --- \| --- \| | \| 9 \|  \|  \|  \| \| --- \| --- \| --- \| --- \| | \| 2 \|  \|  \|  \| \| --- \| --- \| --- \| --- \| | \| 11 \|  \|  \|  \| \| --- \| --- \| --- \| --- \| | \| 1 \| 2 \| 7 \|  \| \| --- \| --- \| --- \| --- \| | \| 2 \| 7 \|  \|  \| \| --- \| --- \| --- \| --- \| | \| 3 \| 4 \|  \|  \| \| --- \| --- \| --- \| --- \| | \| 2 \| 7 \|  \|  \| \| --- \| --- \| --- \| --- \| | \| 1 \| 2 \| 7 \|  \| \| --- \| --- \| --- \| --- \| | \| 10 \|  \|  \|  \| \| --- \| --- \| --- \| --- \| | \| 1 \| 2 \| 7 \|  \| \| --- \| --- \| --- \| --- \| | \| 2 \| 3 \| 4 \| 7 \| \| --- \| --- \| --- \| --- \| |

The changes in the transcription binding sites of understudied HPV-18 isolates compared with the reference sequence NC_001357.

| 0= [NF-1](http://alggen.lsi.upc.es/cgi-bin/promo_v3/promo/promo.cgi?dirDB=TF_8.3&idCon=157683706900&getFile=factors/0.html) | 1= [C/EBP](http://alggen.lsi.upc.es/cgi-bin/promo_v3/promo/promo.cgi?dirDB=TF_8.3&idCon=157683706900&getFile=factors/8.html) α | 2= c-Fos | 3= TFIID | 4= TBP | 5= RXR α | 6= AP-1 | | 7= HMG I(Y) | | 8= c-Jun | 9= POU2F1 | 10= E2F-1 | 11= TEF-1 |
| --- | --- | --- | --- | --- | --- | --- | --- | --- | --- | --- | --- | --- | --- |
| 12= MBF1 | 13=E2F |  |  |  |  |  |  | |  | |  |  |  |

supplementary Table 1b. All the transcription binding sites and factors in the HPV-16 LCR.

|  | 7366 | 7376 | 7396 | 7426 | 7436 | 7456 | 7466 | 7486 | 7506 | 7546 | 7556 | 7576 | 7586 | 7596 | 7616 | 7646 | 7656 | 7666 | 7676 | 7686 | 7706 | 7726 | 7736 | 7766 | 7776 | 7806 | 7816 | 7826 | 7836 | 7846 | 7856 |
| --- | --- | --- | --- | --- | --- | --- | --- | --- | --- | --- | --- | --- | --- | --- | --- | --- | --- | --- | --- | --- | --- | --- | --- | --- | --- | --- | --- | --- | --- | --- | --- |
| NC_001526 | \|  \|  \|  \|  \| \| --- \| --- \| --- \| --- \| | \|  \|  \|  \|  \| \| --- \| --- \| --- \| --- \| | \| 3 \|  \|  \|  \| \| --- \| --- \| --- \| --- \| | \| 0 \|  \|  \|  \| \| --- \| --- \| --- \| --- \| | \| 1 \|  \|  \|  \| \| --- \| --- \| --- \| --- \| | \| 3 \|  \|  \|  \| \| --- \| --- \| --- \| --- \| | \|  \|  \|  \|  \| \| --- \| --- \| --- \| --- \| | \| 3 \|  \|  \|  \| \| --- \| --- \| --- \| --- \| | \| 2 \|  \|  \|  \| \| --- \| --- \| --- \| --- \| | \| 1 \|  \|  \|  \| \| --- \| --- \| --- \| --- \| | \| 3 \|  \|  \|  \| \| --- \| --- \| --- \| --- \| | \| 1 \|  \|  \|  \| \| --- \| --- \| --- \| --- \| | \|  \|  \|  \|  \| \| --- \| --- \| --- \| --- \| | \|  \|  \|  \|  \| \| --- \| --- \| --- \| --- \| | \| 0 \|  \|  \|  \| \| --- \| --- \| --- \| --- \| | \| 4 \|  \|  \|  \| \| --- \| --- \| --- \| --- \| | \|  \|  \|  \|  \| \| --- \| --- \| --- \| --- \| | \| 3 \|  \|  \|  \| \| --- \| --- \| --- \| --- \| | \|  \|  \|  \|  \| \| --- \| --- \| --- \| --- \| | \| 0 \|  \|  \|  \| \| --- \| --- \| --- \| --- \| | \|  \|  \|  \|  \| \| --- \| --- \| --- \| --- \| | \| 0 \| 4 \|  \|  \| \| --- \| --- \| --- \| --- \| | \|  \|  \|  \|  \| \| --- \| --- \| --- \| --- \| | \| 0 \| 4 \|  \|  \| \| --- \| --- \| --- \| --- \| | \| 0 \| 2 \|  \|  \| \| --- \| --- \| --- \| --- \| | \|  \|  \|  \|  \| \| --- \| --- \| --- \| --- \| | \|  \|  \|  \|  \| \| --- \| --- \| --- \| --- \| | \|  \|  \|  \|  \| \| --- \| --- \| --- \| --- \| | \| 1 \| 3 \|  \|  \| \| --- \| --- \| --- \| --- \| | \| 5 \|  \|  \|  \| \| --- \| --- \| --- \| --- \| | \| 4 \|  \|  \|  \| \| --- \| --- \| --- \| --- \| |
| STi13 | \| 1 \|  \|  \|  \| \| --- \| --- \| --- \| --- \| | \| 3 \|  \|  \|  \| \| --- \| --- \| --- \| --- \| | \| 1 \|  \|  \|  \| \| --- \| --- \| --- \| --- \| | \|  \|  \|  \|  \| \| --- \| --- \| --- \| --- \| | \| 0 \|  \|  \|  \| \| --- \| --- \| --- \| --- \| | \|  \|  \|  \|  \| \| --- \| --- \| --- \| --- \| | \| 4 \|  \|  \|  \| \| --- \| --- \| --- \| --- \| | \| 3 \|  \|  \|  \| \| --- \| --- \| --- \| --- \| | \|  \|  \|  \|  \| \| --- \| --- \| --- \| --- \| | \| 0 \| 4 \|  \|  \| \| --- \| --- \| --- \| --- \| | \|  \|  \|  \|  \| \| --- \| --- \| --- \| --- \| | \|  \|  \|  \|  \| \| --- \| --- \| --- \| --- \| | \| 0 \| 4 \|  \|  \| \| --- \| --- \| --- \| --- \| | \| 0 \| 2 \|  \|  \| \| --- \| --- \| --- \| --- \| | \|  \|  \|  \|  \| \| --- \| --- \| --- \| --- \| | \|  \|  \|  \|  \| \| --- \| --- \| --- \| --- \| | \| 1 \| 3 \|  \|  \| \| --- \| --- \| --- \| --- \| | \| 5 \|  \|  \|  \| \| --- \| --- \| --- \| --- \| | \| 4 \|  \|  \|  \| \| --- \| --- \| --- \| --- \| | \|  \|  \|  \|  \| \| --- \| --- \| --- \| --- \| | \| 4 \|  \|  \|  \| \| --- \| --- \| --- \| --- \| | \| 7 \|  \|  \|  \| \| --- \| --- \| --- \| --- \| | \| 4 \|  \|  \|  \| \| --- \| --- \| --- \| --- \| | \| 3 \| 4 \|  \|  \| \| --- \| --- \| --- \| --- \| | \|  \|  \|  \|  \| \| --- \| --- \| --- \| --- \| | \| 6 \|  \|  \|  \| \| --- \| --- \| --- \| --- \| | \| 2 \|  \|  \|  \| \| --- \| --- \| --- \| --- \| | \| 3 \|  \|  \|  \| \| --- \| --- \| --- \| --- \| | \| 0 \|  \|  \|  \| \| --- \| --- \| --- \| --- \| | \|  \|  \|  \|  \| \| --- \| --- \| --- \| --- \| | \|  \|  \|  \|  \| \| --- \| --- \| --- \| --- \| |
| STi20 | \| 1 \|  \|  \|  \| \| --- \| --- \| --- \| --- \| | \| 3 \|  \|  \|  \| \| --- \| --- \| --- \| --- \| | \| 1 \|  \|  \|  \| \| --- \| --- \| --- \| --- \| | \|  \|  \|  \|  \| \| --- \| --- \| --- \| --- \| | \| 0 \|  \|  \|  \| \| --- \| --- \| --- \| --- \| | \|  \|  \|  \|  \| \| --- \| --- \| --- \| --- \| | \| 4 \|  \|  \|  \| \| --- \| --- \| --- \| --- \| | \| 3 \|  \|  \|  \| \| --- \| --- \| --- \| --- \| | \|  \|  \|  \|  \| \| --- \| --- \| --- \| --- \| | \| 0 \| 4 \|  \|  \| \| --- \| --- \| --- \| --- \| | \|  \|  \|  \|  \| \| --- \| --- \| --- \| --- \| | \|  \|  \|  \|  \| \| --- \| --- \| --- \| --- \| | \| 0 \| 4 \|  \|  \| \| --- \| --- \| --- \| --- \| | \| 0 \| 2 \|  \|  \| \| --- \| --- \| --- \| --- \| | \|  \|  \|  \|  \| \| --- \| --- \| --- \| --- \| | \|  \|  \|  \|  \| \| --- \| --- \| --- \| --- \| | \| 1 \| 3 \|  \|  \| \| --- \| --- \| --- \| --- \| | \| 5 \|  \|  \|  \| \| --- \| --- \| --- \| --- \| | \| 4 \|  \|  \|  \| \| --- \| --- \| --- \| --- \| | \|  \|  \|  \|  \| \| --- \| --- \| --- \| --- \| | \| 4 \|  \|  \|  \| \| --- \| --- \| --- \| --- \| | \| 7 \|  \|  \|  \| \| --- \| --- \| --- \| --- \| | \| 4 \|  \|  \|  \| \| --- \| --- \| --- \| --- \| | \| 3 \| 4 \|  \|  \| \| --- \| --- \| --- \| --- \| | \|  \|  \|  \|  \| \| --- \| --- \| --- \| --- \| | \| 6 \|  \|  \|  \| \| --- \| --- \| --- \| --- \| | \| 2 \|  \|  \|  \| \| --- \| --- \| --- \| --- \| | \| 3 \|  \|  \|  \| \| --- \| --- \| --- \| --- \| | \| 0 \|  \|  \|  \| \| --- \| --- \| --- \| --- \| | \|  \|  \|  \|  \| \| --- \| --- \| --- \| --- \| | \|  \|  \|  \|  \| \| --- \| --- \| --- \| --- \| |
| STi21 | \| 1 \|  \|  \|  \| \| --- \| --- \| --- \| --- \| | \| 3 \|  \|  \|  \| \| --- \| --- \| --- \| --- \| | \| 1 \|  \|  \|  \| \| --- \| --- \| --- \| --- \| | \|  \|  \|  \|  \| \| --- \| --- \| --- \| --- \| | \| 0 \|  \|  \|  \| \| --- \| --- \| --- \| --- \| | \|  \|  \|  \|  \| \| --- \| --- \| --- \| --- \| | \| 4 \|  \|  \|  \| \| --- \| --- \| --- \| --- \| | \| 3 \|  \|  \|  \| \| --- \| --- \| --- \| --- \| | \|  \|  \|  \|  \| \| --- \| --- \| --- \| --- \| | \| 0 \| 4 \|  \|  \| \| --- \| --- \| --- \| --- \| | \|  \|  \|  \|  \| \| --- \| --- \| --- \| --- \| | \|  \|  \|  \|  \| \| --- \| --- \| --- \| --- \| | \| 0 \| 4 \|  \|  \| \| --- \| --- \| --- \| --- \| | \| 0 \| 2 \|  \|  \| \| --- \| --- \| --- \| --- \| | \|  \|  \|  \|  \| \| --- \| --- \| --- \| --- \| | \|  \|  \|  \|  \| \| --- \| --- \| --- \| --- \| | \| 1 \| 3 \|  \|  \| \| --- \| --- \| --- \| --- \| | \| 5 \|  \|  \|  \| \| --- \| --- \| --- \| --- \| | \| 4 \|  \|  \|  \| \| --- \| --- \| --- \| --- \| | \|  \|  \|  \|  \| \| --- \| --- \| --- \| --- \| | \| 4 \|  \|  \|  \| \| --- \| --- \| --- \| --- \| | \| 7 \|  \|  \|  \| \| --- \| --- \| --- \| --- \| | \| 4 \|  \|  \|  \| \| --- \| --- \| --- \| --- \| | \| 3 \| 4 \|  \|  \| \| --- \| --- \| --- \| --- \| | \|  \|  \|  \|  \| \| --- \| --- \| --- \| --- \| | \| 6 \|  \|  \|  \| \| --- \| --- \| --- \| --- \| | \| 2 \|  \|  \|  \| \| --- \| --- \| --- \| --- \| | \| 3 \|  \|  \|  \| \| --- \| --- \| --- \| --- \| | \| 0 \|  \|  \|  \| \| --- \| --- \| --- \| --- \| | \|  \|  \|  \|  \| \| --- \| --- \| --- \| --- \| | \|  \|  \|  \|  \| \| --- \| --- \| --- \| --- \| |
| STi26 | \| 1 \|  \|  \|  \| \| --- \| --- \| --- \| --- \| | \| 3 \|  \|  \|  \| \| --- \| --- \| --- \| --- \| | \| 1 \|  \|  \|  \| \| --- \| --- \| --- \| --- \| | \|  \|  \|  \|  \| \| --- \| --- \| --- \| --- \| | \| 0 \|  \|  \|  \| \| --- \| --- \| --- \| --- \| | \|  \|  \|  \|  \| \| --- \| --- \| --- \| --- \| | \| 4 \|  \|  \|  \| \| --- \| --- \| --- \| --- \| | \| 3 \|  \|  \|  \| \| --- \| --- \| --- \| --- \| | \|  \|  \|  \|  \| \| --- \| --- \| --- \| --- \| | \| 0 \| 4 \|  \|  \| \| --- \| --- \| --- \| --- \| | \|  \|  \|  \|  \| \| --- \| --- \| --- \| --- \| | \|  \|  \|  \|  \| \| --- \| --- \| --- \| --- \| | \| 0 \| 4 \|  \|  \| \| --- \| --- \| --- \| --- \| | \| 0 \| 2 \|  \|  \| \| --- \| --- \| --- \| --- \| | \|  \|  \|  \|  \| \| --- \| --- \| --- \| --- \| | \|  \|  \|  \|  \| \| --- \| --- \| --- \| --- \| | \| 1 \| 3 \|  \|  \| \| --- \| --- \| --- \| --- \| | \| 5 \|  \|  \|  \| \| --- \| --- \| --- \| --- \| | \| 4 \|  \|  \|  \| \| --- \| --- \| --- \| --- \| | \|  \|  \|  \|  \| \| --- \| --- \| --- \| --- \| | \| 4 \|  \|  \|  \| \| --- \| --- \| --- \| --- \| | \| 7 \|  \|  \|  \| \| --- \| --- \| --- \| --- \| | \| 4 \|  \|  \|  \| \| --- \| --- \| --- \| --- \| | \| 3 \| 4 \|  \|  \| \| --- \| --- \| --- \| --- \| | \|  \|  \|  \|  \| \| --- \| --- \| --- \| --- \| | \| 6 \|  \|  \|  \| \| --- \| --- \| --- \| --- \| | \| 2 \|  \|  \|  \| \| --- \| --- \| --- \| --- \| | \| 3 \|  \|  \|  \| \| --- \| --- \| --- \| --- \| | \| 0 \|  \|  \|  \| \| --- \| --- \| --- \| --- \| | \|  \|  \|  \|  \| \| --- \| --- \| --- \| --- \| | \|  \|  \|  \|  \| \| --- \| --- \| --- \| --- \| |
| STi27 | \| 1 \|  \|  \|  \| \| --- \| --- \| --- \| --- \| | \| 3 \|  \|  \|  \| \| --- \| --- \| --- \| --- \| | \| 1 \|  \|  \|  \| \| --- \| --- \| --- \| --- \| | \|  \|  \|  \|  \| \| --- \| --- \| --- \| --- \| | \| 0 \|  \|  \|  \| \| --- \| --- \| --- \| --- \| | \|  \|  \|  \|  \| \| --- \| --- \| --- \| --- \| | \| 4 \|  \|  \|  \| \| --- \| --- \| --- \| --- \| | \| 3 \|  \|  \|  \| \| --- \| --- \| --- \| --- \| | \|  \|  \|  \|  \| \| --- \| --- \| --- \| --- \| | \| 0 \| 4 \|  \|  \| \| --- \| --- \| --- \| --- \| | \|  \|  \|  \|  \| \| --- \| --- \| --- \| --- \| | \|  \|  \|  \|  \| \| --- \| --- \| --- \| --- \| | \| 0 \| 4 \|  \|  \| \| --- \| --- \| --- \| --- \| | \| 0 \| 2 \|  \|  \| \| --- \| --- \| --- \| --- \| | \|  \|  \|  \|  \| \| --- \| --- \| --- \| --- \| | \|  \|  \|  \|  \| \| --- \| --- \| --- \| --- \| | \| 1 \| 3 \|  \|  \| \| --- \| --- \| --- \| --- \| | \| 5 \|  \|  \|  \| \| --- \| --- \| --- \| --- \| | \| 4 \|  \|  \|  \| \| --- \| --- \| --- \| --- \| | \|  \|  \|  \|  \| \| --- \| --- \| --- \| --- \| | \| 4 \|  \|  \|  \| \| --- \| --- \| --- \| --- \| | \| 7 \|  \|  \|  \| \| --- \| --- \| --- \| --- \| | \| 4 \|  \|  \|  \| \| --- \| --- \| --- \| --- \| | \| 3 \| 4 \|  \|  \| \| --- \| --- \| --- \| --- \| | \|  \|  \|  \|  \| \| --- \| --- \| --- \| --- \| | \| 6 \|  \|  \|  \| \| --- \| --- \| --- \| --- \| | \| 2 \|  \|  \|  \| \| --- \| --- \| --- \| --- \| | \| 3 \|  \|  \|  \| \| --- \| --- \| --- \| --- \| | \| 0 \|  \|  \|  \| \| --- \| --- \| --- \| --- \| | \|  \|  \|  \|  \| \| --- \| --- \| --- \| --- \| | \|  \|  \|  \|  \| \| --- \| --- \| --- \| --- \| |
| STi28 | \| 1 \|  \|  \|  \| \| --- \| --- \| --- \| --- \| | \| 3 \|  \|  \|  \| \| --- \| --- \| --- \| --- \| | \| 1 \|  \|  \|  \| \| --- \| --- \| --- \| --- \| | \|  \|  \|  \|  \| \| --- \| --- \| --- \| --- \| | \| 0 \|  \|  \|  \| \| --- \| --- \| --- \| --- \| | \|  \|  \|  \|  \| \| --- \| --- \| --- \| --- \| | \| 4 \|  \|  \|  \| \| --- \| --- \| --- \| --- \| | \| 3 \|  \|  \|  \| \| --- \| --- \| --- \| --- \| | \|  \|  \|  \|  \| \| --- \| --- \| --- \| --- \| | \| 0 \| 4 \|  \|  \| \| --- \| --- \| --- \| --- \| | \|  \|  \|  \|  \| \| --- \| --- \| --- \| --- \| | \|  \|  \|  \|  \| \| --- \| --- \| --- \| --- \| | \| 0 \| 4 \|  \|  \| \| --- \| --- \| --- \| --- \| | \| 0 \| 2 \|  \|  \| \| --- \| --- \| --- \| --- \| | \|  \|  \|  \|  \| \| --- \| --- \| --- \| --- \| | \|  \|  \|  \|  \| \| --- \| --- \| --- \| --- \| | \| 1 \| 3 \|  \|  \| \| --- \| --- \| --- \| --- \| | \| 5 \|  \|  \|  \| \| --- \| --- \| --- \| --- \| | \| 4 \|  \|  \|  \| \| --- \| --- \| --- \| --- \| | \|  \|  \|  \|  \| \| --- \| --- \| --- \| --- \| | \| 4 \|  \|  \|  \| \| --- \| --- \| --- \| --- \| | \| 7 \|  \|  \|  \| \| --- \| --- \| --- \| --- \| | \| 4 \|  \|  \|  \| \| --- \| --- \| --- \| --- \| | \| 3 \| 4 \|  \|  \| \| --- \| --- \| --- \| --- \| | \|  \|  \|  \|  \| \| --- \| --- \| --- \| --- \| | \| 6 \|  \|  \|  \| \| --- \| --- \| --- \| --- \| | \| 2 \|  \|  \|  \| \| --- \| --- \| --- \| --- \| | \| 3 \|  \|  \|  \| \| --- \| --- \| --- \| --- \| | \| 0 \|  \|  \|  \| \| --- \| --- \| --- \| --- \| | \|  \|  \|  \|  \| \| --- \| --- \| --- \| --- \| | \|  \|  \|  \|  \| \| --- \| --- \| --- \| --- \| |
| STi38 | \| 1 \|  \|  \|  \| \| --- \| --- \| --- \| --- \| | \| 3 \|  \|  \|  \| \| --- \| --- \| --- \| --- \| | \| 1 \|  \|  \|  \| \| --- \| --- \| --- \| --- \| | \|  \|  \|  \|  \| \| --- \| --- \| --- \| --- \| | \| 0 \|  \|  \|  \| \| --- \| --- \| --- \| --- \| | \|  \|  \|  \|  \| \| --- \| --- \| --- \| --- \| | \| 4 \|  \|  \|  \| \| --- \| --- \| --- \| --- \| | \| 3 \|  \|  \|  \| \| --- \| --- \| --- \| --- \| | \|  \|  \|  \|  \| \| --- \| --- \| --- \| --- \| | \| 0 \| 4 \|  \|  \| \| --- \| --- \| --- \| --- \| | \|  \|  \|  \|  \| \| --- \| --- \| --- \| --- \| | \|  \|  \|  \|  \| \| --- \| --- \| --- \| --- \| | \| 0 \| 4 \|  \|  \| \| --- \| --- \| --- \| --- \| | \| 0 \| 2 \|  \|  \| \| --- \| --- \| --- \| --- \| | \|  \|  \|  \|  \| \| --- \| --- \| --- \| --- \| | \|  \|  \|  \|  \| \| --- \| --- \| --- \| --- \| | \| 1 \| 3 \|  \|  \| \| --- \| --- \| --- \| --- \| | \| 5 \|  \|  \|  \| \| --- \| --- \| --- \| --- \| | \| 4 \|  \|  \|  \| \| --- \| --- \| --- \| --- \| | \|  \|  \|  \|  \| \| --- \| --- \| --- \| --- \| | \| 4 \|  \|  \|  \| \| --- \| --- \| --- \| --- \| | \| 7 \|  \|  \|  \| \| --- \| --- \| --- \| --- \| | \| 4 \|  \|  \|  \| \| --- \| --- \| --- \| --- \| | \| 3 \| 4 \|  \|  \| \| --- \| --- \| --- \| --- \| | \|  \|  \|  \|  \| \| --- \| --- \| --- \| --- \| | \| 6 \|  \|  \|  \| \| --- \| --- \| --- \| --- \| | \| 2 \|  \|  \|  \| \| --- \| --- \| --- \| --- \| | \| 3 \|  \|  \|  \| \| --- \| --- \| --- \| --- \| | \| 0 \|  \|  \|  \| \| --- \| --- \| --- \| --- \| | \|  \|  \|  \|  \| \| --- \| --- \| --- \| --- \| | \|  \|  \|  \|  \| \| --- \| --- \| --- \| --- \| |
| STi62 | \| 1 \|  \|  \|  \| \| --- \| --- \| --- \| --- \| | \| 3 \|  \|  \|  \| \| --- \| --- \| --- \| --- \| | \| 1 \|  \|  \|  \| \| --- \| --- \| --- \| --- \| | \|  \|  \|  \|  \| \| --- \| --- \| --- \| --- \| | \| 0 \|  \|  \|  \| \| --- \| --- \| --- \| --- \| | \|  \|  \|  \|  \| \| --- \| --- \| --- \| --- \| | \| 4 \|  \|  \|  \| \| --- \| --- \| --- \| --- \| | \| 3 \|  \|  \|  \| \| --- \| --- \| --- \| --- \| | \|  \|  \|  \|  \| \| --- \| --- \| --- \| --- \| | \| 0 \| 4 \|  \|  \| \| --- \| --- \| --- \| --- \| | \|  \|  \|  \|  \| \| --- \| --- \| --- \| --- \| | \|  \|  \|  \|  \| \| --- \| --- \| --- \| --- \| | \| 0 \| 4 \|  \|  \| \| --- \| --- \| --- \| --- \| | \| 0 \| 2 \|  \|  \| \| --- \| --- \| --- \| --- \| | \|  \|  \|  \|  \| \| --- \| --- \| --- \| --- \| | \|  \|  \|  \|  \| \| --- \| --- \| --- \| --- \| | \| 1 \| 3 \|  \|  \| \| --- \| --- \| --- \| --- \| | \| 5 \|  \|  \|  \| \| --- \| --- \| --- \| --- \| | \| 4 \|  \|  \|  \| \| --- \| --- \| --- \| --- \| | \|  \|  \|  \|  \| \| --- \| --- \| --- \| --- \| | \| 4 \|  \|  \|  \| \| --- \| --- \| --- \| --- \| | \| 7 \|  \|  \|  \| \| --- \| --- \| --- \| --- \| | \| 4 \|  \|  \|  \| \| --- \| --- \| --- \| --- \| | \| 3 \| 4 \|  \|  \| \| --- \| --- \| --- \| --- \| | \|  \|  \|  \|  \| \| --- \| --- \| --- \| --- \| | \| 6 \|  \|  \|  \| \| --- \| --- \| --- \| --- \| | \| 2 \|  \|  \|  \| \| --- \| --- \| --- \| --- \| | \| 3 \|  \|  \|  \| \| --- \| --- \| --- \| --- \| | \| 0 \|  \|  \|  \| \| --- \| --- \| --- \| --- \| | \|  \|  \|  \|  \| \| --- \| --- \| --- \| --- \| | \|  \|  \|  \|  \| \| --- \| --- \| --- \| --- \| |
| STi65 | \| 1 \|  \|  \|  \| \| --- \| --- \| --- \| --- \| | \| 3 \|  \|  \|  \| \| --- \| --- \| --- \| --- \| | \| 1 \|  \|  \|  \| \| --- \| --- \| --- \| --- \| | \|  \|  \|  \|  \| \| --- \| --- \| --- \| --- \| | \| 0 \|  \|  \|  \| \| --- \| --- \| --- \| --- \| | \|  \|  \|  \|  \| \| --- \| --- \| --- \| --- \| | \| 4 \|  \|  \|  \| \| --- \| --- \| --- \| --- \| | \| 3 \|  \|  \|  \| \| --- \| --- \| --- \| --- \| | \|  \|  \|  \|  \| \| --- \| --- \| --- \| --- \| | \| 0 \| 4 \|  \|  \| \| --- \| --- \| --- \| --- \| | \|  \|  \|  \|  \| \| --- \| --- \| --- \| --- \| | \|  \|  \|  \|  \| \| --- \| --- \| --- \| --- \| | \| 0 \| 4 \|  \|  \| \| --- \| --- \| --- \| --- \| | \| 0 \| 2 \|  \|  \| \| --- \| --- \| --- \| --- \| | \|  \|  \|  \|  \| \| --- \| --- \| --- \| --- \| | \|  \|  \|  \|  \| \| --- \| --- \| --- \| --- \| | \| 1 \| 3 \|  \|  \| \| --- \| --- \| --- \| --- \| | \| 5 \|  \|  \|  \| \| --- \| --- \| --- \| --- \| | \| 4 \|  \|  \|  \| \| --- \| --- \| --- \| --- \| | \|  \|  \|  \|  \| \| --- \| --- \| --- \| --- \| | \| 4 \|  \|  \|  \| \| --- \| --- \| --- \| --- \| | \| 7 \|  \|  \|  \| \| --- \| --- \| --- \| --- \| | \| 4 \|  \|  \|  \| \| --- \| --- \| --- \| --- \| | \| 3 \| 4 \|  \|  \| \| --- \| --- \| --- \| --- \| | \|  \|  \|  \|  \| \| --- \| --- \| --- \| --- \| | \| 6 \|  \|  \|  \| \| --- \| --- \| --- \| --- \| | \| 2 \|  \|  \|  \| \| --- \| --- \| --- \| --- \| | \| 3 \|  \|  \|  \| \| --- \| --- \| --- \| --- \| | \| 0 \|  \|  \|  \| \| --- \| --- \| --- \| --- \| | \|  \|  \|  \|  \| \| --- \| --- \| --- \| --- \| | \|  \|  \|  \|  \| \| --- \| --- \| --- \| --- \| |
| STi101 | \| 1 \|  \|  \|  \| \| --- \| --- \| --- \| --- \| | \| 3 \|  \|  \|  \| \| --- \| --- \| --- \| --- \| | \| 1 \|  \|  \|  \| \| --- \| --- \| --- \| --- \| | \|  \|  \|  \|  \| \| --- \| --- \| --- \| --- \| | \| 0 \|  \|  \|  \| \| --- \| --- \| --- \| --- \| | \|  \|  \|  \|  \| \| --- \| --- \| --- \| --- \| | \| 4 \|  \|  \|  \| \| --- \| --- \| --- \| --- \| | \| 3 \|  \|  \|  \| \| --- \| --- \| --- \| --- \| | \|  \|  \|  \|  \| \| --- \| --- \| --- \| --- \| | \| 0 \| 4 \|  \|  \| \| --- \| --- \| --- \| --- \| | \|  \|  \|  \|  \| \| --- \| --- \| --- \| --- \| | \|  \|  \|  \|  \| \| --- \| --- \| --- \| --- \| | \| 0 \| 4 \|  \|  \| \| --- \| --- \| --- \| --- \| | \| 0 \| 2 \|  \|  \| \| --- \| --- \| --- \| --- \| | \|  \|  \|  \|  \| \| --- \| --- \| --- \| --- \| | \|  \|  \|  \|  \| \| --- \| --- \| --- \| --- \| | \| 1 \| 3 \|  \|  \| \| --- \| --- \| --- \| --- \| | \| 5 \|  \|  \|  \| \| --- \| --- \| --- \| --- \| | \| 4 \|  \|  \|  \| \| --- \| --- \| --- \| --- \| | \|  \|  \|  \|  \| \| --- \| --- \| --- \| --- \| | \| 4 \|  \|  \|  \| \| --- \| --- \| --- \| --- \| | \| 7 \|  \|  \|  \| \| --- \| --- \| --- \| --- \| | \| 4 \|  \|  \|  \| \| --- \| --- \| --- \| --- \| | \| 3 \| 4 \|  \|  \| \| --- \| --- \| --- \| --- \| | \|  \|  \|  \|  \| \| --- \| --- \| --- \| --- \| | \| 6 \|  \|  \|  \| \| --- \| --- \| --- \| --- \| | \| 2 \|  \|  \|  \| \| --- \| --- \| --- \| --- \| | \| 3 \|  \|  \|  \| \| --- \| --- \| --- \| --- \| | \| 0 \|  \|  \|  \| \| --- \| --- \| --- \| --- \| | \|  \|  \|  \|  \| \| --- \| --- \| --- \| --- \| | \|  \|  \|  \|  \| \| --- \| --- \| --- \| --- \| |
| STi106 | \| 1 \|  \|  \|  \| \| --- \| --- \| --- \| --- \| | \| 3 \|  \|  \|  \| \| --- \| --- \| --- \| --- \| | \| 1 \|  \|  \|  \| \| --- \| --- \| --- \| --- \| | \|  \|  \|  \|  \| \| --- \| --- \| --- \| --- \| | \| 0 \|  \|  \|  \| \| --- \| --- \| --- \| --- \| | \|  \|  \|  \|  \| \| --- \| --- \| --- \| --- \| | \| 4 \|  \|  \|  \| \| --- \| --- \| --- \| --- \| | \| 3 \|  \|  \|  \| \| --- \| --- \| --- \| --- \| | \|  \|  \|  \|  \| \| --- \| --- \| --- \| --- \| | \| 0 \| 4 \|  \|  \| \| --- \| --- \| --- \| --- \| | \|  \|  \|  \|  \| \| --- \| --- \| --- \| --- \| | \|  \|  \|  \|  \| \| --- \| --- \| --- \| --- \| | \| 0 \| 4 \|  \|  \| \| --- \| --- \| --- \| --- \| | \| 0 \| 2 \|  \|  \| \| --- \| --- \| --- \| --- \| | \|  \|  \|  \|  \| \| --- \| --- \| --- \| --- \| | \|  \|  \|  \|  \| \| --- \| --- \| --- \| --- \| | \| 1 \| 3 \|  \|  \| \| --- \| --- \| --- \| --- \| | \| 5 \|  \|  \|  \| \| --- \| --- \| --- \| --- \| | \| 4 \|  \|  \|  \| \| --- \| --- \| --- \| --- \| | \|  \|  \|  \|  \| \| --- \| --- \| --- \| --- \| | \| 4 \|  \|  \|  \| \| --- \| --- \| --- \| --- \| | \| 7 \|  \|  \|  \| \| --- \| --- \| --- \| --- \| | \| 4 \|  \|  \|  \| \| --- \| --- \| --- \| --- \| | \| 3 \| 4 \|  \|  \| \| --- \| --- \| --- \| --- \| | \|  \|  \|  \|  \| \| --- \| --- \| --- \| --- \| | \| 6 \|  \|  \|  \| \| --- \| --- \| --- \| --- \| | \| 2 \|  \|  \|  \| \| --- \| --- \| --- \| --- \| | \| 3 \|  \|  \|  \| \| --- \| --- \| --- \| --- \| | \| 0 \|  \|  \|  \| \| --- \| --- \| --- \| --- \| | \|  \|  \|  \|  \| \| --- \| --- \| --- \| --- \| | \|  \|  \|  \|  \| \| --- \| --- \| --- \| --- \| |
| STi110 | \| 1 \|  \|  \|  \| \| --- \| --- \| --- \| --- \| | \| 3 \|  \|  \|  \| \| --- \| --- \| --- \| --- \| | \| 1 \|  \|  \|  \| \| --- \| --- \| --- \| --- \| | \|  \|  \|  \|  \| \| --- \| --- \| --- \| --- \| | \| 0 \|  \|  \|  \| \| --- \| --- \| --- \| --- \| | \|  \|  \|  \|  \| \| --- \| --- \| --- \| --- \| | \| 4 \|  \|  \|  \| \| --- \| --- \| --- \| --- \| | \| 3 \|  \|  \|  \| \| --- \| --- \| --- \| --- \| | \|  \|  \|  \|  \| \| --- \| --- \| --- \| --- \| | \| 0 \| 4 \|  \|  \| \| --- \| --- \| --- \| --- \| | \|  \|  \|  \|  \| \| --- \| --- \| --- \| --- \| | \|  \|  \|  \|  \| \| --- \| --- \| --- \| --- \| | \| 0 \| 4 \|  \|  \| \| --- \| --- \| --- \| --- \| | \| 0 \| 2 \|  \|  \| \| --- \| --- \| --- \| --- \| | \|  \|  \|  \|  \| \| --- \| --- \| --- \| --- \| | \|  \|  \|  \|  \| \| --- \| --- \| --- \| --- \| | \| 1 \| 3 \|  \|  \| \| --- \| --- \| --- \| --- \| | \| 5 \|  \|  \|  \| \| --- \| --- \| --- \| --- \| | \| 4 \|  \|  \|  \| \| --- \| --- \| --- \| --- \| | \|  \|  \|  \|  \| \| --- \| --- \| --- \| --- \| | \| 4 \|  \|  \|  \| \| --- \| --- \| --- \| --- \| | \| 7 \|  \|  \|  \| \| --- \| --- \| --- \| --- \| | \| 4 \|  \|  \|  \| \| --- \| --- \| --- \| --- \| | \| 3 \| 4 \|  \|  \| \| --- \| --- \| --- \| --- \| | \|  \|  \|  \|  \| \| --- \| --- \| --- \| --- \| | \| 6 \|  \|  \|  \| \| --- \| --- \| --- \| --- \| | \| 2 \|  \|  \|  \| \| --- \| --- \| --- \| --- \| | \| 3 \|  \|  \|  \| \| --- \| --- \| --- \| --- \| | \| 0 \|  \|  \|  \| \| --- \| --- \| --- \| --- \| | \|  \|  \|  \|  \| \| --- \| --- \| --- \| --- \| | \|  \|  \|  \|  \| \| --- \| --- \| --- \| --- \| |
| TP86 | \| 1 \|  \|  \|  \| \| --- \| --- \| --- \| --- \| | \| 3 \|  \|  \|  \| \| --- \| --- \| --- \| --- \| | \| 1 \|  \|  \|  \| \| --- \| --- \| --- \| --- \| | \|  \|  \|  \|  \| \| --- \| --- \| --- \| --- \| | \| 0 \|  \|  \|  \| \| --- \| --- \| --- \| --- \| | \|  \|  \|  \|  \| \| --- \| --- \| --- \| --- \| | \| 4 \|  \|  \|  \| \| --- \| --- \| --- \| --- \| | \| 3 \|  \|  \|  \| \| --- \| --- \| --- \| --- \| | \|  \|  \|  \|  \| \| --- \| --- \| --- \| --- \| | \| 0 \| 4 \|  \|  \| \| --- \| --- \| --- \| --- \| | \|  \|  \|  \|  \| \| --- \| --- \| --- \| --- \| | \|  \|  \|  \|  \| \| --- \| --- \| --- \| --- \| | \| 0 \| 4 \|  \|  \| \| --- \| --- \| --- \| --- \| | \| 0 \| 2 \|  \|  \| \| --- \| --- \| --- \| --- \| | \|  \|  \|  \|  \| \| --- \| --- \| --- \| --- \| | \|  \|  \|  \|  \| \| --- \| --- \| --- \| --- \| | \| 1 \| 3 \|  \|  \| \| --- \| --- \| --- \| --- \| | \| 5 \|  \|  \|  \| \| --- \| --- \| --- \| --- \| | \| 4 \|  \|  \|  \| \| --- \| --- \| --- \| --- \| | \|  \|  \|  \|  \| \| --- \| --- \| --- \| --- \| | \| 4 \|  \|  \|  \| \| --- \| --- \| --- \| --- \| | \| 7 \|  \|  \|  \| \| --- \| --- \| --- \| --- \| | \| 4 \|  \|  \|  \| \| --- \| --- \| --- \| --- \| | \| 3 \| 4 \|  \|  \| \| --- \| --- \| --- \| --- \| | \|  \|  \|  \|  \| \| --- \| --- \| --- \| --- \| | \| 6 \|  \|  \|  \| \| --- \| --- \| --- \| --- \| | \|  \|  \|  \|  \| \| --- \| --- \| --- \| --- \| | \| 3 \|  \|  \|  \| \| --- \| --- \| --- \| --- \| | \| 0 \|  \|  \|  \| \| --- \| --- \| --- \| --- \| | \|  \|  \|  \|  \| \| --- \| --- \| --- \| --- \| | \|  \|  \|  \|  \| \| --- \| --- \| --- \| --- \| |
| TP128 | \| 1 \|  \|  \|  \| \| --- \| --- \| --- \| --- \| | \| 3 \|  \|  \|  \| \| --- \| --- \| --- \| --- \| | \| 1 \|  \|  \|  \| \| --- \| --- \| --- \| --- \| | \|  \|  \|  \|  \| \| --- \| --- \| --- \| --- \| | \| 0 \|  \|  \|  \| \| --- \| --- \| --- \| --- \| | \|  \|  \|  \|  \| \| --- \| --- \| --- \| --- \| | \| 4 \|  \|  \|  \| \| --- \| --- \| --- \| --- \| | \| 3 \|  \|  \|  \| \| --- \| --- \| --- \| --- \| | \|  \|  \|  \|  \| \| --- \| --- \| --- \| --- \| | \| 0 \| 4 \|  \|  \| \| --- \| --- \| --- \| --- \| | \|  \|  \|  \|  \| \| --- \| --- \| --- \| --- \| | \|  \|  \|  \|  \| \| --- \| --- \| --- \| --- \| | \| 0 \| 4 \|  \|  \| \| --- \| --- \| --- \| --- \| | \| 0 \| 2 \|  \|  \| \| --- \| --- \| --- \| --- \| | \|  \|  \|  \|  \| \| --- \| --- \| --- \| --- \| | \|  \|  \|  \|  \| \| --- \| --- \| --- \| --- \| | \| 1 \| 3 \|  \|  \| \| --- \| --- \| --- \| --- \| | \| 5 \|  \|  \|  \| \| --- \| --- \| --- \| --- \| | \| 4 \|  \|  \|  \| \| --- \| --- \| --- \| --- \| | \|  \|  \|  \|  \| \| --- \| --- \| --- \| --- \| | \| 4 \|  \|  \|  \| \| --- \| --- \| --- \| --- \| | \| 7 \|  \|  \|  \| \| --- \| --- \| --- \| --- \| | \| 4 \|  \|  \|  \| \| --- \| --- \| --- \| --- \| | \| 3 \| 4 \|  \|  \| \| --- \| --- \| --- \| --- \| | \|  \|  \|  \|  \| \| --- \| --- \| --- \| --- \| | \| 6 \|  \|  \|  \| \| --- \| --- \| --- \| --- \| | \| 2 \|  \|  \|  \| \| --- \| --- \| --- \| --- \| | \| 3 \|  \|  \|  \| \| --- \| --- \| --- \| --- \| | \| 0 \|  \|  \|  \| \| --- \| --- \| --- \| --- \| | \|  \|  \|  \|  \| \| --- \| --- \| --- \| --- \| | \|  \|  \|  \|  \| \| --- \| --- \| --- \| --- \| |
| TP130 | \| 1 \|  \|  \|  \| \| --- \| --- \| --- \| --- \| | \| 3 \|  \|  \|  \| \| --- \| --- \| --- \| --- \| | \| 1 \|  \|  \|  \| \| --- \| --- \| --- \| --- \| | \|  \|  \|  \|  \| \| --- \| --- \| --- \| --- \| | \| 0 \|  \|  \|  \| \| --- \| --- \| --- \| --- \| | \|  \|  \|  \|  \| \| --- \| --- \| --- \| --- \| | \| 4 \|  \|  \|  \| \| --- \| --- \| --- \| --- \| | \| 3 \|  \|  \|  \| \| --- \| --- \| --- \| --- \| | \|  \|  \|  \|  \| \| --- \| --- \| --- \| --- \| | \| 0 \| 4 \|  \|  \| \| --- \| --- \| --- \| --- \| | \|  \|  \|  \|  \| \| --- \| --- \| --- \| --- \| | \|  \|  \|  \|  \| \| --- \| --- \| --- \| --- \| | \| 0 \| 4 \|  \|  \| \| --- \| --- \| --- \| --- \| | \| 0 \| 2 \|  \|  \| \| --- \| --- \| --- \| --- \| | \|  \|  \|  \|  \| \| --- \| --- \| --- \| --- \| | \|  \|  \|  \|  \| \| --- \| --- \| --- \| --- \| | \| 1 \| 3 \|  \|  \| \| --- \| --- \| --- \| --- \| | \| 5 \|  \|  \|  \| \| --- \| --- \| --- \| --- \| | \| 4 \|  \|  \|  \| \| --- \| --- \| --- \| --- \| | \|  \|  \|  \|  \| \| --- \| --- \| --- \| --- \| | \| 4 \|  \|  \|  \| \| --- \| --- \| --- \| --- \| | \| 7 \|  \|  \|  \| \| --- \| --- \| --- \| --- \| | \| 4 \|  \|  \|  \| \| --- \| --- \| --- \| --- \| | \| 3 \| 4 \|  \|  \| \| --- \| --- \| --- \| --- \| | \|  \|  \|  \|  \| \| --- \| --- \| --- \| --- \| | \| 6 \|  \|  \|  \| \| --- \| --- \| --- \| --- \| | \| 2 \|  \|  \|  \| \| --- \| --- \| --- \| --- \| | \| 3 \|  \|  \|  \| \| --- \| --- \| --- \| --- \| | \| 0 \|  \|  \|  \| \| --- \| --- \| --- \| --- \| | \|  \|  \|  \|  \| \| --- \| --- \| --- \| --- \| | \|  \|  \|  \|  \| \| --- \| --- \| --- \| --- \| |
| TP228 | \| 1 \|  \|  \|  \| \| --- \| --- \| --- \| --- \| | \| 3 \|  \|  \|  \| \| --- \| --- \| --- \| --- \| | \| 1 \|  \|  \|  \| \| --- \| --- \| --- \| --- \| | \|  \|  \|  \|  \| \| --- \| --- \| --- \| --- \| | \| 0 \|  \|  \|  \| \| --- \| --- \| --- \| --- \| | \|  \|  \|  \|  \| \| --- \| --- \| --- \| --- \| | \| 4 \|  \|  \|  \| \| --- \| --- \| --- \| --- \| | \| 3 \|  \|  \|  \| \| --- \| --- \| --- \| --- \| | \| 0 \|  \|  \|  \| \| --- \| --- \| --- \| --- \| | \| 0 \| 4 \|  \|  \| \| --- \| --- \| --- \| --- \| | \|  \|  \|  \|  \| \| --- \| --- \| --- \| --- \| | \|  \|  \|  \|  \| \| --- \| --- \| --- \| --- \| | \| 0 \| 4 \|  \|  \| \| --- \| --- \| --- \| --- \| | \| 0 \| 2 \|  \|  \| \| --- \| --- \| --- \| --- \| | \|  \|  \|  \|  \| \| --- \| --- \| --- \| --- \| | \|  \|  \|  \|  \| \| --- \| --- \| --- \| --- \| | \| 1 \| 3 \|  \|  \| \| --- \| --- \| --- \| --- \| | \| 5 \|  \|  \|  \| \| --- \| --- \| --- \| --- \| | \| 4 \|  \|  \|  \| \| --- \| --- \| --- \| --- \| | \|  \|  \|  \|  \| \| --- \| --- \| --- \| --- \| | \| 4 \|  \|  \|  \| \| --- \| --- \| --- \| --- \| | \| 7 \|  \|  \|  \| \| --- \| --- \| --- \| --- \| | \| 4 \|  \|  \|  \| \| --- \| --- \| --- \| --- \| | \| 3 \| 4 \|  \|  \| \| --- \| --- \| --- \| --- \| | \|  \|  \|  \|  \| \| --- \| --- \| --- \| --- \| | \| 6 \|  \|  \|  \| \| --- \| --- \| --- \| --- \| | \| 2 \|  \|  \|  \| \| --- \| --- \| --- \| --- \| | \| 3 \|  \|  \|  \| \| --- \| --- \| --- \| --- \| | \| 0 \|  \|  \|  \| \| --- \| --- \| --- \| --- \| | \|  \|  \|  \|  \| \| --- \| --- \| --- \| --- \| | \|  \|  \|  \|  \| \| --- \| --- \| --- \| --- \| |
| TP542 | \| 1 \|  \|  \|  \| \| --- \| --- \| --- \| --- \| | \| 3 \|  \|  \|  \| \| --- \| --- \| --- \| --- \| | \| 1 \|  \|  \|  \| \| --- \| --- \| --- \| --- \| | \|  \|  \|  \|  \| \| --- \| --- \| --- \| --- \| | \| 0 \|  \|  \|  \| \| --- \| --- \| --- \| --- \| | \|  \|  \|  \|  \| \| --- \| --- \| --- \| --- \| | \| 4 \|  \|  \|  \| \| --- \| --- \| --- \| --- \| | \| 3 \|  \|  \|  \| \| --- \| --- \| --- \| --- \| | \|  \|  \|  \|  \| \| --- \| --- \| --- \| --- \| | \| 0 \| 4 \|  \|  \| \| --- \| --- \| --- \| --- \| | \|  \|  \|  \|  \| \| --- \| --- \| --- \| --- \| | \|  \|  \|  \|  \| \| --- \| --- \| --- \| --- \| | \| 0 \| 4 \|  \|  \| \| --- \| --- \| --- \| --- \| | \| 0 \| 2 \|  \|  \| \| --- \| --- \| --- \| --- \| | \|  \|  \|  \|  \| \| --- \| --- \| --- \| --- \| | \|  \|  \|  \|  \| \| --- \| --- \| --- \| --- \| | \| 1 \| 3 \|  \|  \| \| --- \| --- \| --- \| --- \| | \| 5 \|  \|  \|  \| \| --- \| --- \| --- \| --- \| | \| 4 \|  \|  \|  \| \| --- \| --- \| --- \| --- \| | \|  \|  \|  \|  \| \| --- \| --- \| --- \| --- \| | \| 4 \|  \|  \|  \| \| --- \| --- \| --- \| --- \| | \| 7 \|  \|  \|  \| \| --- \| --- \| --- \| --- \| | \| 4 \|  \|  \|  \| \| --- \| --- \| --- \| --- \| | \| 3 \| 4 \|  \|  \| \| --- \| --- \| --- \| --- \| | \|  \|  \|  \|  \| \| --- \| --- \| --- \| --- \| | \| 6 \|  \|  \|  \| \| --- \| --- \| --- \| --- \| | \| 2 \|  \|  \|  \| \| --- \| --- \| --- \| --- \| | \| 3 \|  \|  \|  \| \| --- \| --- \| --- \| --- \| | \| 0 \|  \|  \|  \| \| --- \| --- \| --- \| --- \| | \|  \|  \|  \|  \| \| --- \| --- \| --- \| --- \| | \|  \|  \|  \|  \| \| --- \| --- \| --- \| --- \| |
| TP2067 | \| 1 \|  \|  \|  \| \| --- \| --- \| --- \| --- \| | \| 3 \|  \|  \|  \| \| --- \| --- \| --- \| --- \| | \| 1 \|  \|  \|  \| \| --- \| --- \| --- \| --- \| | \|  \|  \|  \|  \| \| --- \| --- \| --- \| --- \| | \| 0 \|  \|  \|  \| \| --- \| --- \| --- \| --- \| | \|  \|  \|  \|  \| \| --- \| --- \| --- \| --- \| | \| 4 \|  \|  \|  \| \| --- \| --- \| --- \| --- \| | \| 3 \|  \|  \|  \| \| --- \| --- \| --- \| --- \| | \|  \|  \|  \|  \| \| --- \| --- \| --- \| --- \| | \| 0 \| 4 \|  \|  \| \| --- \| --- \| --- \| --- \| | \|  \|  \|  \|  \| \| --- \| --- \| --- \| --- \| | \|  \|  \|  \|  \| \| --- \| --- \| --- \| --- \| | \| 0 \| 4 \|  \|  \| \| --- \| --- \| --- \| --- \| | \| 0 \| 2 \|  \|  \| \| --- \| --- \| --- \| --- \| | \|  \|  \|  \|  \| \| --- \| --- \| --- \| --- \| | \|  \|  \|  \|  \| \| --- \| --- \| --- \| --- \| | \| 1 \| 3 \|  \|  \| \| --- \| --- \| --- \| --- \| | \| 5 \|  \|  \|  \| \| --- \| --- \| --- \| --- \| | \| 4 \|  \|  \|  \| \| --- \| --- \| --- \| --- \| | \|  \|  \|  \|  \| \| --- \| --- \| --- \| --- \| | \| 4 \|  \|  \|  \| \| --- \| --- \| --- \| --- \| | \| 7 \|  \|  \|  \| \| --- \| --- \| --- \| --- \| | \| 4 \|  \|  \|  \| \| --- \| --- \| --- \| --- \| | \| 3 \| 4 \|  \|  \| \| --- \| --- \| --- \| --- \| | \|  \|  \|  \|  \| \| --- \| --- \| --- \| --- \| | \| 6 \|  \|  \|  \| \| --- \| --- \| --- \| --- \| | \| 2 \|  \|  \|  \| \| --- \| --- \| --- \| --- \| | \| 3 \|  \|  \|  \| \| --- \| --- \| --- \| --- \| | \| 0 \|  \|  \|  \| \| --- \| --- \| --- \| --- \| | \|  \|  \|  \|  \| \| --- \| --- \| --- \| --- \| | \|  \|  \|  \|  \| \| --- \| --- \| --- \| --- \| |
| TP2198 | \| 1 \|  \|  \|  \| \| --- \| --- \| --- \| --- \| | \| 3 \|  \|  \|  \| \| --- \| --- \| --- \| --- \| | \| 1 \|  \|  \|  \| \| --- \| --- \| --- \| --- \| | \|  \|  \|  \|  \| \| --- \| --- \| --- \| --- \| | \| 0 \|  \|  \|  \| \| --- \| --- \| --- \| --- \| | \|  \|  \|  \|  \| \| --- \| --- \| --- \| --- \| | \| 4 \|  \|  \|  \| \| --- \| --- \| --- \| --- \| | \| 3 \|  \|  \|  \| \| --- \| --- \| --- \| --- \| | \|  \|  \|  \|  \| \| --- \| --- \| --- \| --- \| | \| 0 \| 4 \|  \|  \| \| --- \| --- \| --- \| --- \| | \|  \|  \|  \|  \| \| --- \| --- \| --- \| --- \| | \|  \|  \|  \|  \| \| --- \| --- \| --- \| --- \| | \| 0 \| 4 \|  \|  \| \| --- \| --- \| --- \| --- \| | \| 0 \| 2 \|  \|  \| \| --- \| --- \| --- \| --- \| | \|  \|  \|  \|  \| \| --- \| --- \| --- \| --- \| | \|  \|  \|  \|  \| \| --- \| --- \| --- \| --- \| | \| 1 \| 3 \|  \|  \| \| --- \| --- \| --- \| --- \| | \| 5 \|  \|  \|  \| \| --- \| --- \| --- \| --- \| | \| 4 \|  \|  \|  \| \| --- \| --- \| --- \| --- \| | \|  \|  \|  \|  \| \| --- \| --- \| --- \| --- \| | \| 4 \|  \|  \|  \| \| --- \| --- \| --- \| --- \| | \| 7 \|  \|  \|  \| \| --- \| --- \| --- \| --- \| | \| 4 \|  \|  \|  \| \| --- \| --- \| --- \| --- \| | \| 3 \| 4 \|  \|  \| \| --- \| --- \| --- \| --- \| | \|  \|  \|  \|  \| \| --- \| --- \| --- \| --- \| | \| 6 \|  \|  \|  \| \| --- \| --- \| --- \| --- \| | \| 2 \|  \|  \|  \| \| --- \| --- \| --- \| --- \| | \| 3 \|  \|  \|  \| \| --- \| --- \| --- \| --- \| | \| 0 \|  \|  \|  \| \| --- \| --- \| --- \| --- \| | \|  \|  \|  \|  \| \| --- \| --- \| --- \| --- \| | \|  \|  \|  \|  \| \| --- \| --- \| --- \| --- \| |
| TP2219 | \| 1 \|  \|  \|  \| \| --- \| --- \| --- \| --- \| | \| 3 \|  \|  \|  \| \| --- \| --- \| --- \| --- \| | \| 1 \|  \|  \|  \| \| --- \| --- \| --- \| --- \| | \|  \|  \|  \|  \| \| --- \| --- \| --- \| --- \| | \| 0 \|  \|  \|  \| \| --- \| --- \| --- \| --- \| | \|  \|  \|  \|  \| \| --- \| --- \| --- \| --- \| | \| 4 \|  \|  \|  \| \| --- \| --- \| --- \| --- \| | \| 3 \|  \|  \|  \| \| --- \| --- \| --- \| --- \| | \|  \|  \|  \|  \| \| --- \| --- \| --- \| --- \| | \| 0 \| 4 \|  \|  \| \| --- \| --- \| --- \| --- \| | \|  \|  \|  \|  \| \| --- \| --- \| --- \| --- \| | \|  \|  \|  \|  \| \| --- \| --- \| --- \| --- \| | \| 0 \| 4 \|  \|  \| \| --- \| --- \| --- \| --- \| | \| 0 \| 2 \|  \|  \| \| --- \| --- \| --- \| --- \| | \|  \|  \|  \|  \| \| --- \| --- \| --- \| --- \| | \|  \|  \|  \|  \| \| --- \| --- \| --- \| --- \| | \| 1 \| 3 \|  \|  \| \| --- \| --- \| --- \| --- \| | \| 5 \|  \|  \|  \| \| --- \| --- \| --- \| --- \| | \| 4 \|  \|  \|  \| \| --- \| --- \| --- \| --- \| | \|  \|  \|  \|  \| \| --- \| --- \| --- \| --- \| | \| 4 \|  \|  \|  \| \| --- \| --- \| --- \| --- \| | \| 7 \|  \|  \|  \| \| --- \| --- \| --- \| --- \| | \| 4 \|  \|  \|  \| \| --- \| --- \| --- \| --- \| | \| 3 \| 4 \|  \|  \| \| --- \| --- \| --- \| --- \| | \|  \|  \|  \|  \| \| --- \| --- \| --- \| --- \| | \| 6 \|  \|  \|  \| \| --- \| --- \| --- \| --- \| | \| 2 \|  \|  \|  \| \| --- \| --- \| --- \| --- \| | \| 3 \|  \|  \|  \| \| --- \| --- \| --- \| --- \| | \| 0 \|  \|  \|  \| \| --- \| --- \| --- \| --- \| | \|  \|  \|  \|  \| \| --- \| --- \| --- \| --- \| | \|  \|  \|  \|  \| \| --- \| --- \| --- \| --- \| |
| TP2249 | \| 1 \|  \|  \|  \| \| --- \| --- \| --- \| --- \| | \| 3 \|  \|  \|  \| \| --- \| --- \| --- \| --- \| | \| 1 \|  \|  \|  \| \| --- \| --- \| --- \| --- \| | \|  \|  \|  \|  \| \| --- \| --- \| --- \| --- \| | \| 0 \|  \|  \|  \| \| --- \| --- \| --- \| --- \| | \|  \|  \|  \|  \| \| --- \| --- \| --- \| --- \| | \| 4 \|  \|  \|  \| \| --- \| --- \| --- \| --- \| | \| 3 \|  \|  \|  \| \| --- \| --- \| --- \| --- \| | \|  \|  \|  \|  \| \| --- \| --- \| --- \| --- \| | \| 0 \| 4 \|  \|  \| \| --- \| --- \| --- \| --- \| | \|  \|  \|  \|  \| \| --- \| --- \| --- \| --- \| | \|  \|  \|  \|  \| \| --- \| --- \| --- \| --- \| | \| 0 \| 4 \|  \|  \| \| --- \| --- \| --- \| --- \| | \| 0 \| 2 \|  \|  \| \| --- \| --- \| --- \| --- \| | \|  \|  \|  \|  \| \| --- \| --- \| --- \| --- \| | \|  \|  \|  \|  \| \| --- \| --- \| --- \| --- \| | \| 1 \| 3 \|  \|  \| \| --- \| --- \| --- \| --- \| | \| 5 \|  \|  \|  \| \| --- \| --- \| --- \| --- \| | \| 4 \|  \|  \|  \| \| --- \| --- \| --- \| --- \| | \|  \|  \|  \|  \| \| --- \| --- \| --- \| --- \| | \| 4 \|  \|  \|  \| \| --- \| --- \| --- \| --- \| | \| 7 \|  \|  \|  \| \| --- \| --- \| --- \| --- \| | \| 4 \|  \|  \|  \| \| --- \| --- \| --- \| --- \| | \| 3 \| 4 \|  \|  \| \| --- \| --- \| --- \| --- \| | \|  \|  \|  \|  \| \| --- \| --- \| --- \| --- \| | \| 6 \|  \|  \|  \| \| --- \| --- \| --- \| --- \| | \| 2 \|  \|  \|  \| \| --- \| --- \| --- \| --- \| | \| 3 \|  \|  \|  \| \| --- \| --- \| --- \| --- \| | \| 0 \|  \|  \|  \| \| --- \| --- \| --- \| --- \| | \|  \|  \|  \|  \| \| --- \| --- \| --- \| --- \| | \|  \|  \|  \|  \| \| --- \| --- \| --- \| --- \| |
| TP2325 | \| 1 \|  \|  \|  \| \| --- \| --- \| --- \| --- \| | \| 3 \|  \|  \|  \| \| --- \| --- \| --- \| --- \| | \| 1 \|  \|  \|  \| \| --- \| --- \| --- \| --- \| | \|  \|  \|  \|  \| \| --- \| --- \| --- \| --- \| | \| 0 \|  \|  \|  \| \| --- \| --- \| --- \| --- \| | \|  \|  \|  \|  \| \| --- \| --- \| --- \| --- \| | \| 4 \|  \|  \|  \| \| --- \| --- \| --- \| --- \| | \| 3 \|  \|  \|  \| \| --- \| --- \| --- \| --- \| | \|  \|  \|  \|  \| \| --- \| --- \| --- \| --- \| | \| 0 \| 4 \|  \|  \| \| --- \| --- \| --- \| --- \| | \|  \|  \|  \|  \| \| --- \| --- \| --- \| --- \| | \|  \|  \|  \|  \| \| --- \| --- \| --- \| --- \| | \| 0 \| 4 \|  \|  \| \| --- \| --- \| --- \| --- \| | \| 0 \| 2 \|  \|  \| \| --- \| --- \| --- \| --- \| | \|  \|  \|  \|  \| \| --- \| --- \| --- \| --- \| | \|  \|  \|  \|  \| \| --- \| --- \| --- \| --- \| | \| 1 \| 3 \|  \|  \| \| --- \| --- \| --- \| --- \| | \| 5 \|  \|  \|  \| \| --- \| --- \| --- \| --- \| | \| 4 \|  \|  \|  \| \| --- \| --- \| --- \| --- \| | \|  \|  \|  \|  \| \| --- \| --- \| --- \| --- \| | \| 4 \|  \|  \|  \| \| --- \| --- \| --- \| --- \| | \| 7 \|  \|  \|  \| \| --- \| --- \| --- \| --- \| | \| 4 \|  \|  \|  \| \| --- \| --- \| --- \| --- \| | \| 3 \| 4 \|  \|  \| \| --- \| --- \| --- \| --- \| | \|  \|  \|  \|  \| \| --- \| --- \| --- \| --- \| | \| 6 \|  \|  \|  \| \| --- \| --- \| --- \| --- \| | \| 2 \|  \|  \|  \| \| --- \| --- \| --- \| --- \| | \| 3 \|  \|  \|  \| \| --- \| --- \| --- \| --- \| | \| 0 \|  \|  \|  \| \| --- \| --- \| --- \| --- \| | \|  \|  \|  \|  \| \| --- \| --- \| --- \| --- \| | \|  \|  \|  \|  \| \| --- \| --- \| --- \| --- \| |
| TP2790 | \| 1 \|  \|  \|  \| \| --- \| --- \| --- \| --- \| | \| 3 \|  \|  \|  \| \| --- \| --- \| --- \| --- \| | \| 1 \|  \|  \|  \| \| --- \| --- \| --- \| --- \| | \|  \|  \|  \|  \| \| --- \| --- \| --- \| --- \| | \| 0 \|  \|  \|  \| \| --- \| --- \| --- \| --- \| | \|  \|  \|  \|  \| \| --- \| --- \| --- \| --- \| | \| 4 \|  \|  \|  \| \| --- \| --- \| --- \| --- \| | \| 3 \|  \|  \|  \| \| --- \| --- \| --- \| --- \| | \|  \|  \|  \|  \| \| --- \| --- \| --- \| --- \| | \| 0 \| 4 \|  \|  \| \| --- \| --- \| --- \| --- \| | \|  \|  \|  \|  \| \| --- \| --- \| --- \| --- \| | \|  \|  \|  \|  \| \| --- \| --- \| --- \| --- \| | \| 0 \| 4 \|  \|  \| \| --- \| --- \| --- \| --- \| | \| 0 \| 2 \|  \|  \| \| --- \| --- \| --- \| --- \| | \|  \|  \|  \|  \| \| --- \| --- \| --- \| --- \| | \|  \|  \|  \|  \| \| --- \| --- \| --- \| --- \| | \| 1 \| 3 \|  \|  \| \| --- \| --- \| --- \| --- \| | \| 5 \|  \|  \|  \| \| --- \| --- \| --- \| --- \| | \| 4 \|  \|  \|  \| \| --- \| --- \| --- \| --- \| | \|  \|  \|  \|  \| \| --- \| --- \| --- \| --- \| | \| 4 \|  \|  \|  \| \| --- \| --- \| --- \| --- \| | \| 7 \|  \|  \|  \| \| --- \| --- \| --- \| --- \| | \| 4 \|  \|  \|  \| \| --- \| --- \| --- \| --- \| | \| 3 \| 4 \|  \|  \| \| --- \| --- \| --- \| --- \| | \|  \|  \|  \|  \| \| --- \| --- \| --- \| --- \| | \| 6 \|  \|  \|  \| \| --- \| --- \| --- \| --- \| | \| 2 \|  \|  \|  \| \| --- \| --- \| --- \| --- \| | \| 3 \|  \|  \|  \| \| --- \| --- \| --- \| --- \| | \| 0 \|  \|  \|  \| \| --- \| --- \| --- \| --- \| | \|  \|  \|  \|  \| \| --- \| --- \| --- \| --- \| | \|  \|  \|  \|  \| \| --- \| --- \| --- \| --- \| |
| TP3144 | \| 1 \|  \|  \|  \| \| --- \| --- \| --- \| --- \| | \| 3 \|  \|  \|  \| \| --- \| --- \| --- \| --- \| | \| 1 \|  \|  \|  \| \| --- \| --- \| --- \| --- \| | \|  \|  \|  \|  \| \| --- \| --- \| --- \| --- \| | \| 0 \|  \|  \|  \| \| --- \| --- \| --- \| --- \| | \|  \|  \|  \|  \| \| --- \| --- \| --- \| --- \| | \| 4 \|  \|  \|  \| \| --- \| --- \| --- \| --- \| | \| 3 \|  \|  \|  \| \| --- \| --- \| --- \| --- \| | \|  \|  \|  \|  \| \| --- \| --- \| --- \| --- \| | \| 0 \| 4 \|  \|  \| \| --- \| --- \| --- \| --- \| | \|  \|  \|  \|  \| \| --- \| --- \| --- \| --- \| | \|  \|  \|  \|  \| \| --- \| --- \| --- \| --- \| | \| 0 \| 4 \|  \|  \| \| --- \| --- \| --- \| --- \| | \| 0 \| 2 \|  \|  \| \| --- \| --- \| --- \| --- \| | \|  \|  \|  \|  \| \| --- \| --- \| --- \| --- \| | \|  \|  \|  \|  \| \| --- \| --- \| --- \| --- \| | \| 1 \| 3 \|  \|  \| \| --- \| --- \| --- \| --- \| | \| 5 \|  \|  \|  \| \| --- \| --- \| --- \| --- \| | \| 4 \|  \|  \|  \| \| --- \| --- \| --- \| --- \| | \|  \|  \|  \|  \| \| --- \| --- \| --- \| --- \| | \| 4 \|  \|  \|  \| \| --- \| --- \| --- \| --- \| | \| 7 \|  \|  \|  \| \| --- \| --- \| --- \| --- \| | \| 4 \|  \|  \|  \| \| --- \| --- \| --- \| --- \| | \| 3 \| 4 \|  \|  \| \| --- \| --- \| --- \| --- \| | \|  \|  \|  \|  \| \| --- \| --- \| --- \| --- \| | \| 6 \|  \|  \|  \| \| --- \| --- \| --- \| --- \| | \|  \|  \|  \|  \| \| --- \| --- \| --- \| --- \| | \| 3 \|  \|  \|  \| \| --- \| --- \| --- \| --- \| | \| 0 \|  \|  \|  \| \| --- \| --- \| --- \| --- \| | \|  \|  \|  \|  \| \| --- \| --- \| --- \| --- \| | \|  \|  \|  \|  \| \| --- \| --- \| --- \| --- \| |
| TP3376 | \| 1 \|  \|  \|  \| \| --- \| --- \| --- \| --- \| | \| 3 \|  \|  \|  \| \| --- \| --- \| --- \| --- \| | \| 1 \|  \|  \|  \| \| --- \| --- \| --- \| --- \| | \|  \|  \|  \|  \| \| --- \| --- \| --- \| --- \| | \| 0 \|  \|  \|  \| \| --- \| --- \| --- \| --- \| | \|  \|  \|  \|  \| \| --- \| --- \| --- \| --- \| | \| 4 \|  \|  \|  \| \| --- \| --- \| --- \| --- \| | \| 3 \|  \|  \|  \| \| --- \| --- \| --- \| --- \| | \|  \|  \|  \|  \| \| --- \| --- \| --- \| --- \| | \| 0 \| 4 \|  \|  \| \| --- \| --- \| --- \| --- \| | \|  \|  \|  \|  \| \| --- \| --- \| --- \| --- \| | \|  \|  \|  \|  \| \| --- \| --- \| --- \| --- \| | \| 0 \| 4 \|  \|  \| \| --- \| --- \| --- \| --- \| | \| 0 \| 2 \|  \|  \| \| --- \| --- \| --- \| --- \| | \|  \|  \|  \|  \| \| --- \| --- \| --- \| --- \| | \|  \|  \|  \|  \| \| --- \| --- \| --- \| --- \| | \| 1 \| 3 \|  \|  \| \| --- \| --- \| --- \| --- \| | \| 5 \|  \|  \|  \| \| --- \| --- \| --- \| --- \| | \| 4 \|  \|  \|  \| \| --- \| --- \| --- \| --- \| | \|  \|  \|  \|  \| \| --- \| --- \| --- \| --- \| | \| 4 \|  \|  \|  \| \| --- \| --- \| --- \| --- \| | \| 7 \|  \|  \|  \| \| --- \| --- \| --- \| --- \| | \| 4 \|  \|  \|  \| \| --- \| --- \| --- \| --- \| | \| 3 \| 4 \|  \|  \| \| --- \| --- \| --- \| --- \| | \|  \|  \|  \|  \| \| --- \| --- \| --- \| --- \| | \| 6 \|  \|  \|  \| \| --- \| --- \| --- \| --- \| | \| 2 \|  \|  \|  \| \| --- \| --- \| --- \| --- \| | \| 3 \|  \|  \|  \| \| --- \| --- \| --- \| --- \| | \| 0 \|  \|  \|  \| \| --- \| --- \| --- \| --- \| | \|  \|  \|  \|  \| \| --- \| --- \| --- \| --- \| | \|  \|  \|  \|  \| \| --- \| --- \| --- \| --- \| |
| TP3700 | \| 1 \|  \|  \|  \| \| --- \| --- \| --- \| --- \| | \| 3 \|  \|  \|  \| \| --- \| --- \| --- \| --- \| | \| 1 \|  \|  \|  \| \| --- \| --- \| --- \| --- \| | \|  \|  \|  \|  \| \| --- \| --- \| --- \| --- \| | \| 0 \|  \|  \|  \| \| --- \| --- \| --- \| --- \| | \|  \|  \|  \|  \| \| --- \| --- \| --- \| --- \| | \| 4 \|  \|  \|  \| \| --- \| --- \| --- \| --- \| | \| 3 \|  \|  \|  \| \| --- \| --- \| --- \| --- \| | \|  \|  \|  \|  \| \| --- \| --- \| --- \| --- \| | \| 0 \| 4 \|  \|  \| \| --- \| --- \| --- \| --- \| | \|  \|  \|  \|  \| \| --- \| --- \| --- \| --- \| | \|  \|  \|  \|  \| \| --- \| --- \| --- \| --- \| | \| 0 \| 4 \|  \|  \| \| --- \| --- \| --- \| --- \| | \| 0 \| 2 \|  \|  \| \| --- \| --- \| --- \| --- \| | \|  \|  \|  \|  \| \| --- \| --- \| --- \| --- \| | \|  \|  \|  \|  \| \| --- \| --- \| --- \| --- \| | \| 1 \| 3 \|  \|  \| \| --- \| --- \| --- \| --- \| | \| 5 \|  \|  \|  \| \| --- \| --- \| --- \| --- \| | \| 4 \|  \|  \|  \| \| --- \| --- \| --- \| --- \| | \|  \|  \|  \|  \| \| --- \| --- \| --- \| --- \| | \| 4 \|  \|  \|  \| \| --- \| --- \| --- \| --- \| | \| 7 \|  \|  \|  \| \| --- \| --- \| --- \| --- \| | \| 4 \|  \|  \|  \| \| --- \| --- \| --- \| --- \| | \| 3 \| 4 \|  \|  \| \| --- \| --- \| --- \| --- \| | \|  \|  \|  \|  \| \| --- \| --- \| --- \| --- \| | \| 6 \|  \|  \|  \| \| --- \| --- \| --- \| --- \| | \| 2 \|  \|  \|  \| \| --- \| --- \| --- \| --- \| | \| 3 \|  \|  \|  \| \| --- \| --- \| --- \| --- \| | \| 0 \|  \|  \|  \| \| --- \| --- \| --- \| --- \| | \|  \|  \|  \|  \| \| --- \| --- \| --- \| --- \| | \|  \|  \|  \|  \| \| --- \| --- \| --- \| --- \| |
| TP3888 | \| 1 \|  \|  \|  \| \| --- \| --- \| --- \| --- \| | \| 3 \|  \|  \|  \| \| --- \| --- \| --- \| --- \| | \| 1 \|  \|  \|  \| \| --- \| --- \| --- \| --- \| | \|  \|  \|  \|  \| \| --- \| --- \| --- \| --- \| | \| 0 \|  \|  \|  \| \| --- \| --- \| --- \| --- \| | \|  \|  \|  \|  \| \| --- \| --- \| --- \| --- \| | \| 4 \|  \|  \|  \| \| --- \| --- \| --- \| --- \| | \| 3 \|  \|  \|  \| \| --- \| --- \| --- \| --- \| | \|  \|  \|  \|  \| \| --- \| --- \| --- \| --- \| | \| 0 \| 4 \|  \|  \| \| --- \| --- \| --- \| --- \| | \|  \|  \|  \|  \| \| --- \| --- \| --- \| --- \| | \|  \|  \|  \|  \| \| --- \| --- \| --- \| --- \| | \| 0 \| 4 \|  \|  \| \| --- \| --- \| --- \| --- \| | \| 0 \| 2 \|  \|  \| \| --- \| --- \| --- \| --- \| | \|  \|  \|  \|  \| \| --- \| --- \| --- \| --- \| | \|  \|  \|  \|  \| \| --- \| --- \| --- \| --- \| | \| 1 \| 3 \|  \|  \| \| --- \| --- \| --- \| --- \| | \| 5 \|  \|  \|  \| \| --- \| --- \| --- \| --- \| | \| 4 \|  \|  \|  \| \| --- \| --- \| --- \| --- \| | \|  \|  \|  \|  \| \| --- \| --- \| --- \| --- \| | \| 4 \|  \|  \|  \| \| --- \| --- \| --- \| --- \| | \| 7 \|  \|  \|  \| \| --- \| --- \| --- \| --- \| | \| 4 \|  \|  \|  \| \| --- \| --- \| --- \| --- \| | \| 3 \| 4 \|  \|  \| \| --- \| --- \| --- \| --- \| | \|  \|  \|  \|  \| \| --- \| --- \| --- \| --- \| | \| 6 \|  \|  \|  \| \| --- \| --- \| --- \| --- \| | \| 2 \|  \|  \|  \| \| --- \| --- \| --- \| --- \| | \| 3 \|  \|  \|  \| \| --- \| --- \| --- \| --- \| | \| 0 \|  \|  \|  \| \| --- \| --- \| --- \| --- \| | \|  \|  \|  \|  \| \| --- \| --- \| --- \| --- \| | \|  \|  \|  \|  \| \| --- \| --- \| --- \| --- \| |
| TP3992 | \| 1 \|  \|  \|  \| \| --- \| --- \| --- \| --- \| | \| 3 \|  \|  \|  \| \| --- \| --- \| --- \| --- \| | \| 1 \|  \|  \|  \| \| --- \| --- \| --- \| --- \| | \|  \|  \|  \|  \| \| --- \| --- \| --- \| --- \| | \| 0 \|  \|  \|  \| \| --- \| --- \| --- \| --- \| | \|  \|  \|  \|  \| \| --- \| --- \| --- \| --- \| | \| 4 \|  \|  \|  \| \| --- \| --- \| --- \| --- \| | \| 3 \|  \|  \|  \| \| --- \| --- \| --- \| --- \| | \|  \|  \|  \|  \| \| --- \| --- \| --- \| --- \| | \| 0 \| 4 \|  \|  \| \| --- \| --- \| --- \| --- \| | \|  \|  \|  \|  \| \| --- \| --- \| --- \| --- \| | \|  \|  \|  \|  \| \| --- \| --- \| --- \| --- \| | \| 0 \| 4 \|  \|  \| \| --- \| --- \| --- \| --- \| | \| 0 \| 2 \|  \|  \| \| --- \| --- \| --- \| --- \| | \|  \|  \|  \|  \| \| --- \| --- \| --- \| --- \| | \|  \|  \|  \|  \| \| --- \| --- \| --- \| --- \| | \| 1 \| 3 \|  \|  \| \| --- \| --- \| --- \| --- \| | \| 5 \|  \|  \|  \| \| --- \| --- \| --- \| --- \| | \| 4 \|  \|  \|  \| \| --- \| --- \| --- \| --- \| | \|  \|  \|  \|  \| \| --- \| --- \| --- \| --- \| | \| 4 \|  \|  \|  \| \| --- \| --- \| --- \| --- \| | \| 7 \|  \|  \|  \| \| --- \| --- \| --- \| --- \| | \| 4 \|  \|  \|  \| \| --- \| --- \| --- \| --- \| | \| 3 \| 4 \|  \|  \| \| --- \| --- \| --- \| --- \| | \|  \|  \|  \|  \| \| --- \| --- \| --- \| --- \| | \| 6 \|  \|  \|  \| \| --- \| --- \| --- \| --- \| | \| 2 \|  \|  \|  \| \| --- \| --- \| --- \| --- \| | \| 3 \|  \|  \|  \| \| --- \| --- \| --- \| --- \| | \| 0 \|  \|  \|  \| \| --- \| --- \| --- \| --- \| | \|  \|  \|  \|  \| \| --- \| --- \| --- \| --- \| | \|  \|  \|  \|  \| \| --- \| --- \| --- \| --- \| |
| TP4088 | \| 1 \|  \|  \|  \| \| --- \| --- \| --- \| --- \| | \| 3 \|  \|  \|  \| \| --- \| --- \| --- \| --- \| | \| 1 \|  \|  \|  \| \| --- \| --- \| --- \| --- \| | \|  \|  \|  \|  \| \| --- \| --- \| --- \| --- \| | \| 0 \|  \|  \|  \| \| --- \| --- \| --- \| --- \| | \|  \|  \|  \|  \| \| --- \| --- \| --- \| --- \| | \| 4 \|  \|  \|  \| \| --- \| --- \| --- \| --- \| | \| 3 \|  \|  \|  \| \| --- \| --- \| --- \| --- \| | \|  \|  \|  \|  \| \| --- \| --- \| --- \| --- \| | \| 0 \| 4 \|  \|  \| \| --- \| --- \| --- \| --- \| | \|  \|  \|  \|  \| \| --- \| --- \| --- \| --- \| | \|  \|  \|  \|  \| \| --- \| --- \| --- \| --- \| | \| 0 \| 4 \|  \|  \| \| --- \| --- \| --- \| --- \| | \| 0 \| 2 \|  \|  \| \| --- \| --- \| --- \| --- \| | \|  \|  \|  \|  \| \| --- \| --- \| --- \| --- \| | \|  \|  \|  \|  \| \| --- \| --- \| --- \| --- \| | \| 1 \| 3 \|  \|  \| \| --- \| --- \| --- \| --- \| | \| 5 \|  \|  \|  \| \| --- \| --- \| --- \| --- \| | \| 4 \|  \|  \|  \| \| --- \| --- \| --- \| --- \| | \|  \|  \|  \|  \| \| --- \| --- \| --- \| --- \| | \| 4 \|  \|  \|  \| \| --- \| --- \| --- \| --- \| | \| 7 \|  \|  \|  \| \| --- \| --- \| --- \| --- \| | \| 4 \|  \|  \|  \| \| --- \| --- \| --- \| --- \| | \| 3 \| 4 \|  \|  \| \| --- \| --- \| --- \| --- \| | \|  \|  \|  \|  \| \| --- \| --- \| --- \| --- \| | \| 6 \|  \|  \|  \| \| --- \| --- \| --- \| --- \| | \|  \|  \|  \|  \| \| --- \| --- \| --- \| --- \| | \| 3 \|  \|  \|  \| \| --- \| --- \| --- \| --- \| | \| 0 \|  \|  \|  \| \| --- \| --- \| --- \| --- \| | \|  \|  \|  \|  \| \| --- \| --- \| --- \| --- \| | \|  \|  \|  \|  \| \| --- \| --- \| --- \| --- \| |
| TP4450 | \| 1 \|  \|  \|  \| \| --- \| --- \| --- \| --- \| | \| 3 \|  \|  \|  \| \| --- \| --- \| --- \| --- \| | \| 1 \|  \|  \|  \| \| --- \| --- \| --- \| --- \| | \|  \|  \|  \|  \| \| --- \| --- \| --- \| --- \| | \| 0 \|  \|  \|  \| \| --- \| --- \| --- \| --- \| | \|  \|  \|  \|  \| \| --- \| --- \| --- \| --- \| | \| 4 \|  \|  \|  \| \| --- \| --- \| --- \| --- \| | \| 3 \|  \|  \|  \| \| --- \| --- \| --- \| --- \| | \|  \|  \|  \|  \| \| --- \| --- \| --- \| --- \| | \| 0 \| 4 \|  \|  \| \| --- \| --- \| --- \| --- \| | \|  \|  \|  \|  \| \| --- \| --- \| --- \| --- \| | \|  \|  \|  \|  \| \| --- \| --- \| --- \| --- \| | \| 0 \| 4 \|  \|  \| \| --- \| --- \| --- \| --- \| | \| 0 \| 2 \|  \|  \| \| --- \| --- \| --- \| --- \| | \|  \|  \|  \|  \| \| --- \| --- \| --- \| --- \| | \|  \|  \|  \|  \| \| --- \| --- \| --- \| --- \| | \| 1 \| 3 \|  \|  \| \| --- \| --- \| --- \| --- \| | \| 5 \|  \|  \|  \| \| --- \| --- \| --- \| --- \| | \| 4 \|  \|  \|  \| \| --- \| --- \| --- \| --- \| | \|  \|  \|  \|  \| \| --- \| --- \| --- \| --- \| | \| 4 \|  \|  \|  \| \| --- \| --- \| --- \| --- \| | \| 7 \|  \|  \|  \| \| --- \| --- \| --- \| --- \| | \| 4 \|  \|  \|  \| \| --- \| --- \| --- \| --- \| | \| 3 \| 4 \|  \|  \| \| --- \| --- \| --- \| --- \| | \|  \|  \|  \|  \| \| --- \| --- \| --- \| --- \| | \| 6 \|  \|  \|  \| \| --- \| --- \| --- \| --- \| | \| 2 \|  \|  \|  \| \| --- \| --- \| --- \| --- \| | \| 3 \|  \|  \|  \| \| --- \| --- \| --- \| --- \| | \| 0 \|  \|  \|  \| \| --- \| --- \| --- \| --- \| | \|  \|  \|  \|  \| \| --- \| --- \| --- \| --- \| | \|  \|  \|  \|  \| \| --- \| --- \| --- \| --- \| |
| TP4494 | \| 1 \|  \|  \|  \| \| --- \| --- \| --- \| --- \| | \| 3 \|  \|  \|  \| \| --- \| --- \| --- \| --- \| | \| 1 \|  \|  \|  \| \| --- \| --- \| --- \| --- \| | \|  \|  \|  \|  \| \| --- \| --- \| --- \| --- \| | \| 0 \|  \|  \|  \| \| --- \| --- \| --- \| --- \| | \|  \|  \|  \|  \| \| --- \| --- \| --- \| --- \| | \| 4 \|  \|  \|  \| \| --- \| --- \| --- \| --- \| | \| 3 \|  \|  \|  \| \| --- \| --- \| --- \| --- \| | \|  \|  \|  \|  \| \| --- \| --- \| --- \| --- \| | \| 0 \| 4 \|  \|  \| \| --- \| --- \| --- \| --- \| | \|  \|  \|  \|  \| \| --- \| --- \| --- \| --- \| | \|  \|  \|  \|  \| \| --- \| --- \| --- \| --- \| | \| 0 \| 4 \|  \|  \| \| --- \| --- \| --- \| --- \| | \| 0 \| 2 \|  \|  \| \| --- \| --- \| --- \| --- \| | \|  \|  \|  \|  \| \| --- \| --- \| --- \| --- \| | \|  \|  \|  \|  \| \| --- \| --- \| --- \| --- \| | \| 1 \| 3 \|  \|  \| \| --- \| --- \| --- \| --- \| | \| 5 \|  \|  \|  \| \| --- \| --- \| --- \| --- \| | \| 4 \|  \|  \|  \| \| --- \| --- \| --- \| --- \| | \|  \|  \|  \|  \| \| --- \| --- \| --- \| --- \| | \| 4 \|  \|  \|  \| \| --- \| --- \| --- \| --- \| | \| 7 \|  \|  \|  \| \| --- \| --- \| --- \| --- \| | \| 4 \|  \|  \|  \| \| --- \| --- \| --- \| --- \| | \| 3 \| 4 \|  \|  \| \| --- \| --- \| --- \| --- \| | \|  \|  \|  \|  \| \| --- \| --- \| --- \| --- \| | \| 6 \|  \|  \|  \| \| --- \| --- \| --- \| --- \| | \| 2 \|  \|  \|  \| \| --- \| --- \| --- \| --- \| | \| 3 \|  \|  \|  \| \| --- \| --- \| --- \| --- \| | \| 0 \|  \|  \|  \| \| --- \| --- \| --- \| --- \| | \|  \|  \|  \|  \| \| --- \| --- \| --- \| --- \| | \|  \|  \|  \|  \| \| --- \| --- \| --- \| --- \| |
| TP4822 | \| 1 \|  \|  \|  \| \| --- \| --- \| --- \| --- \| | \| 3 \|  \|  \|  \| \| --- \| --- \| --- \| --- \| | \| 1 \|  \|  \|  \| \| --- \| --- \| --- \| --- \| | \|  \|  \|  \|  \| \| --- \| --- \| --- \| --- \| | \| 0 \|  \|  \|  \| \| --- \| --- \| --- \| --- \| | \|  \|  \|  \|  \| \| --- \| --- \| --- \| --- \| | \| 4 \|  \|  \|  \| \| --- \| --- \| --- \| --- \| | \| 3 \|  \|  \|  \| \| --- \| --- \| --- \| --- \| | \|  \|  \|  \|  \| \| --- \| --- \| --- \| --- \| | \| 0 \| 4 \|  \|  \| \| --- \| --- \| --- \| --- \| | \|  \|  \|  \|  \| \| --- \| --- \| --- \| --- \| | \|  \|  \|  \|  \| \| --- \| --- \| --- \| --- \| | \| 0 \| 4 \|  \|  \| \| --- \| --- \| --- \| --- \| | \| 0 \| 2 \|  \|  \| \| --- \| --- \| --- \| --- \| | \|  \|  \|  \|  \| \| --- \| --- \| --- \| --- \| | \|  \|  \|  \|  \| \| --- \| --- \| --- \| --- \| | \| 1 \| 3 \|  \|  \| \| --- \| --- \| --- \| --- \| | \| 5 \|  \|  \|  \| \| --- \| --- \| --- \| --- \| | \| 4 \|  \|  \|  \| \| --- \| --- \| --- \| --- \| | \|  \|  \|  \|  \| \| --- \| --- \| --- \| --- \| | \| 4 \|  \|  \|  \| \| --- \| --- \| --- \| --- \| | \| 7 \|  \|  \|  \| \| --- \| --- \| --- \| --- \| | \| 4 \|  \|  \|  \| \| --- \| --- \| --- \| --- \| | \| 3 \| 4 \|  \|  \| \| --- \| --- \| --- \| --- \| | \|  \|  \|  \|  \| \| --- \| --- \| --- \| --- \| | \| 6 \|  \|  \|  \| \| --- \| --- \| --- \| --- \| | \| 2 \|  \|  \|  \| \| --- \| --- \| --- \| --- \| | \| 3 \|  \|  \|  \| \| --- \| --- \| --- \| --- \| | \| 0 \|  \|  \|  \| \| --- \| --- \| --- \| --- \| | \|  \|  \|  \|  \| \| --- \| --- \| --- \| --- \| | \|  \|  \|  \|  \| \| --- \| --- \| --- \| --- \| |
| TP4904 | \| 1 \|  \|  \|  \| \| --- \| --- \| --- \| --- \| | \| 3 \|  \|  \|  \| \| --- \| --- \| --- \| --- \| | \| 1 \|  \|  \|  \| \| --- \| --- \| --- \| --- \| | \|  \|  \|  \|  \| \| --- \| --- \| --- \| --- \| | \| 0 \|  \|  \|  \| \| --- \| --- \| --- \| --- \| | \|  \|  \|  \|  \| \| --- \| --- \| --- \| --- \| | \| 4 \|  \|  \|  \| \| --- \| --- \| --- \| --- \| | \| 3 \|  \|  \|  \| \| --- \| --- \| --- \| --- \| | \|  \|  \|  \|  \| \| --- \| --- \| --- \| --- \| | \| 0 \| 4 \|  \|  \| \| --- \| --- \| --- \| --- \| | \|  \|  \|  \|  \| \| --- \| --- \| --- \| --- \| | \|  \|  \|  \|  \| \| --- \| --- \| --- \| --- \| | \| 0 \| 4 \|  \|  \| \| --- \| --- \| --- \| --- \| | \| 0 \| 2 \|  \|  \| \| --- \| --- \| --- \| --- \| | \|  \|  \|  \|  \| \| --- \| --- \| --- \| --- \| | \|  \|  \|  \|  \| \| --- \| --- \| --- \| --- \| | \| 1 \| 3 \|  \|  \| \| --- \| --- \| --- \| --- \| | \| 5 \|  \|  \|  \| \| --- \| --- \| --- \| --- \| | \| 4 \|  \|  \|  \| \| --- \| --- \| --- \| --- \| | \|  \|  \|  \|  \| \| --- \| --- \| --- \| --- \| | \| 4 \|  \|  \|  \| \| --- \| --- \| --- \| --- \| | \| 7 \|  \|  \|  \| \| --- \| --- \| --- \| --- \| | \| 4 \|  \|  \|  \| \| --- \| --- \| --- \| --- \| | \| 3 \| 4 \|  \|  \| \| --- \| --- \| --- \| --- \| | \|  \|  \|  \|  \| \| --- \| --- \| --- \| --- \| | \| 6 \|  \|  \|  \| \| --- \| --- \| --- \| --- \| | \| 2 \|  \|  \|  \| \| --- \| --- \| --- \| --- \| | \| 3 \|  \|  \|  \| \| --- \| --- \| --- \| --- \| | \| 0 \|  \|  \|  \| \| --- \| --- \| --- \| --- \| | \|  \|  \|  \|  \| \| --- \| --- \| --- \| --- \| | \|  \|  \|  \|  \| \| --- \| --- \| --- \| --- \| |
| TP4938 | \| 1 \|  \|  \|  \| \| --- \| --- \| --- \| --- \| | \| 3 \|  \|  \|  \| \| --- \| --- \| --- \| --- \| | \| 1 \|  \|  \|  \| \| --- \| --- \| --- \| --- \| | \|  \|  \|  \|  \| \| --- \| --- \| --- \| --- \| | \| 0 \|  \|  \|  \| \| --- \| --- \| --- \| --- \| | \|  \|  \|  \|  \| \| --- \| --- \| --- \| --- \| | \| 4 \|  \|  \|  \| \| --- \| --- \| --- \| --- \| | \| 3 \|  \|  \|  \| \| --- \| --- \| --- \| --- \| | \|  \|  \|  \|  \| \| --- \| --- \| --- \| --- \| | \| 0 \| 4 \|  \|  \| \| --- \| --- \| --- \| --- \| | \|  \|  \|  \|  \| \| --- \| --- \| --- \| --- \| | \|  \|  \|  \|  \| \| --- \| --- \| --- \| --- \| | \| 0 \| 4 \|  \|  \| \| --- \| --- \| --- \| --- \| | \| 0 \| 2 \|  \|  \| \| --- \| --- \| --- \| --- \| | \|  \|  \|  \|  \| \| --- \| --- \| --- \| --- \| | \|  \|  \|  \|  \| \| --- \| --- \| --- \| --- \| | \| 1 \| 3 \|  \|  \| \| --- \| --- \| --- \| --- \| | \| 5 \|  \|  \|  \| \| --- \| --- \| --- \| --- \| | \| 4 \|  \|  \|  \| \| --- \| --- \| --- \| --- \| | \|  \|  \|  \|  \| \| --- \| --- \| --- \| --- \| | \| 4 \|  \|  \|  \| \| --- \| --- \| --- \| --- \| | \| 7 \|  \|  \|  \| \| --- \| --- \| --- \| --- \| | \| 4 \|  \|  \|  \| \| --- \| --- \| --- \| --- \| | \| 3 \| 4 \|  \|  \| \| --- \| --- \| --- \| --- \| | \|  \|  \|  \|  \| \| --- \| --- \| --- \| --- \| | \| 6 \|  \|  \|  \| \| --- \| --- \| --- \| --- \| | \|  \|  \|  \|  \| \| --- \| --- \| --- \| --- \| | \| 3 \|  \|  \|  \| \| --- \| --- \| --- \| --- \| | \| 0 \|  \|  \|  \| \| --- \| --- \| --- \| --- \| | \|  \|  \|  \|  \| \| --- \| --- \| --- \| --- \| | \|  \|  \|  \|  \| \| --- \| --- \| --- \| --- \| |
| TP5124 | \| 1 \|  \|  \|  \| \| --- \| --- \| --- \| --- \| | \| 3 \|  \|  \|  \| \| --- \| --- \| --- \| --- \| | \| 1 \|  \|  \|  \| \| --- \| --- \| --- \| --- \| | \|  \|  \|  \|  \| \| --- \| --- \| --- \| --- \| | \| 0 \|  \|  \|  \| \| --- \| --- \| --- \| --- \| | \|  \|  \|  \|  \| \| --- \| --- \| --- \| --- \| | \| 4 \|  \|  \|  \| \| --- \| --- \| --- \| --- \| | \| 3 \|  \|  \|  \| \| --- \| --- \| --- \| --- \| | \|  \|  \|  \|  \| \| --- \| --- \| --- \| --- \| | \| 0 \| 4 \|  \|  \| \| --- \| --- \| --- \| --- \| | \|  \|  \|  \|  \| \| --- \| --- \| --- \| --- \| | \|  \|  \|  \|  \| \| --- \| --- \| --- \| --- \| | \| 0 \| 4 \|  \|  \| \| --- \| --- \| --- \| --- \| | \| 0 \| 2 \|  \|  \| \| --- \| --- \| --- \| --- \| | \|  \|  \|  \|  \| \| --- \| --- \| --- \| --- \| | \|  \|  \|  \|  \| \| --- \| --- \| --- \| --- \| | \| 1 \| 3 \|  \|  \| \| --- \| --- \| --- \| --- \| | \| 5 \|  \|  \|  \| \| --- \| --- \| --- \| --- \| | \| 4 \|  \|  \|  \| \| --- \| --- \| --- \| --- \| | \|  \|  \|  \|  \| \| --- \| --- \| --- \| --- \| | \| 4 \|  \|  \|  \| \| --- \| --- \| --- \| --- \| | \| 7 \|  \|  \|  \| \| --- \| --- \| --- \| --- \| | \| 4 \|  \|  \|  \| \| --- \| --- \| --- \| --- \| | \| 3 \| 4 \|  \|  \| \| --- \| --- \| --- \| --- \| | \|  \|  \|  \|  \| \| --- \| --- \| --- \| --- \| | \| 6 \|  \|  \|  \| \| --- \| --- \| --- \| --- \| | \| 2 \|  \|  \|  \| \| --- \| --- \| --- \| --- \| | \| 3 \|  \|  \|  \| \| --- \| --- \| --- \| --- \| | \| 0 \|  \|  \|  \| \| --- \| --- \| --- \| --- \| | \|  \|  \|  \|  \| \| --- \| --- \| --- \| --- \| | \|  \|  \|  \|  \| \| --- \| --- \| --- \| --- \| |
| TP5210 | \| 1 \|  \|  \|  \| \| --- \| --- \| --- \| --- \| | \| 3 \|  \|  \|  \| \| --- \| --- \| --- \| --- \| | \| 1 \|  \|  \|  \| \| --- \| --- \| --- \| --- \| | \|  \|  \|  \|  \| \| --- \| --- \| --- \| --- \| | \| 0 \|  \|  \|  \| \| --- \| --- \| --- \| --- \| | \|  \|  \|  \|  \| \| --- \| --- \| --- \| --- \| | \| 4 \|  \|  \|  \| \| --- \| --- \| --- \| --- \| | \| 3 \|  \|  \|  \| \| --- \| --- \| --- \| --- \| | \|  \|  \|  \|  \| \| --- \| --- \| --- \| --- \| | \| 0 \| 4 \|  \|  \| \| --- \| --- \| --- \| --- \| | \|  \|  \|  \|  \| \| --- \| --- \| --- \| --- \| | \|  \|  \|  \|  \| \| --- \| --- \| --- \| --- \| | \| 0 \| 4 \|  \|  \| \| --- \| --- \| --- \| --- \| | \| 0 \| 2 \|  \|  \| \| --- \| --- \| --- \| --- \| | \|  \|  \|  \|  \| \| --- \| --- \| --- \| --- \| | \|  \|  \|  \|  \| \| --- \| --- \| --- \| --- \| | \| 1 \| 3 \|  \|  \| \| --- \| --- \| --- \| --- \| | \| 5 \|  \|  \|  \| \| --- \| --- \| --- \| --- \| | \| 4 \|  \|  \|  \| \| --- \| --- \| --- \| --- \| | \|  \|  \|  \|  \| \| --- \| --- \| --- \| --- \| | \| 4 \|  \|  \|  \| \| --- \| --- \| --- \| --- \| | \| 7 \|  \|  \|  \| \| --- \| --- \| --- \| --- \| | \| 4 \|  \|  \|  \| \| --- \| --- \| --- \| --- \| | \| 3 \| 4 \|  \|  \| \| --- \| --- \| --- \| --- \| | \|  \|  \|  \|  \| \| --- \| --- \| --- \| --- \| | \| 6 \|  \|  \|  \| \| --- \| --- \| --- \| --- \| | \| 2 \|  \|  \|  \| \| --- \| --- \| --- \| --- \| | \| 3 \|  \|  \|  \| \| --- \| --- \| --- \| --- \| | \| 0 \|  \|  \|  \| \| --- \| --- \| --- \| --- \| | \|  \|  \|  \|  \| \| --- \| --- \| --- \| --- \| | \|  \|  \|  \|  \| \| --- \| --- \| --- \| --- \| |
| TP5246 | \| 1 \|  \|  \|  \| \| --- \| --- \| --- \| --- \| | \| 3 \|  \|  \|  \| \| --- \| --- \| --- \| --- \| | \| 1 \|  \|  \|  \| \| --- \| --- \| --- \| --- \| | \|  \|  \|  \|  \| \| --- \| --- \| --- \| --- \| | \| 0 \|  \|  \|  \| \| --- \| --- \| --- \| --- \| | \|  \|  \|  \|  \| \| --- \| --- \| --- \| --- \| | \| 4 \|  \|  \|  \| \| --- \| --- \| --- \| --- \| | \| 3 \|  \|  \|  \| \| --- \| --- \| --- \| --- \| | \|  \|  \|  \|  \| \| --- \| --- \| --- \| --- \| | \| 0 \| 4 \|  \|  \| \| --- \| --- \| --- \| --- \| | \|  \|  \|  \|  \| \| --- \| --- \| --- \| --- \| | \|  \|  \|  \|  \| \| --- \| --- \| --- \| --- \| | \| 0 \| 4 \|  \|  \| \| --- \| --- \| --- \| --- \| | \| 0 \| 2 \|  \|  \| \| --- \| --- \| --- \| --- \| | \|  \|  \|  \|  \| \| --- \| --- \| --- \| --- \| | \|  \|  \|  \|  \| \| --- \| --- \| --- \| --- \| | \| 1 \| 3 \|  \|  \| \| --- \| --- \| --- \| --- \| | \| 5 \|  \|  \|  \| \| --- \| --- \| --- \| --- \| | \| 4 \|  \|  \|  \| \| --- \| --- \| --- \| --- \| | \|  \|  \|  \|  \| \| --- \| --- \| --- \| --- \| | \| 4 \|  \|  \|  \| \| --- \| --- \| --- \| --- \| | \| 7 \|  \|  \|  \| \| --- \| --- \| --- \| --- \| | \| 4 \|  \|  \|  \| \| --- \| --- \| --- \| --- \| | \| 3 \| 4 \|  \|  \| \| --- \| --- \| --- \| --- \| | \|  \|  \|  \|  \| \| --- \| --- \| --- \| --- \| | \| 6 \|  \|  \|  \| \| --- \| --- \| --- \| --- \| | \| 2 \|  \|  \|  \| \| --- \| --- \| --- \| --- \| | \| 3 \|  \|  \|  \| \| --- \| --- \| --- \| --- \| | \| 0 \|  \|  \|  \| \| --- \| --- \| --- \| --- \| | \|  \|  \|  \|  \| \| --- \| --- \| --- \| --- \| | \|  \|  \|  \|  \| \| --- \| --- \| --- \| --- \| |
| TP5304 | \| 1 \|  \|  \|  \| \| --- \| --- \| --- \| --- \| | \| 3 \|  \|  \|  \| \| --- \| --- \| --- \| --- \| | \| 1 \|  \|  \|  \| \| --- \| --- \| --- \| --- \| | \|  \|  \|  \|  \| \| --- \| --- \| --- \| --- \| | \| 0 \|  \|  \|  \| \| --- \| --- \| --- \| --- \| | \|  \|  \|  \|  \| \| --- \| --- \| --- \| --- \| | \| 4 \|  \|  \|  \| \| --- \| --- \| --- \| --- \| | \| 3 \|  \|  \|  \| \| --- \| --- \| --- \| --- \| | \|  \|  \|  \|  \| \| --- \| --- \| --- \| --- \| | \| 0 \| 4 \|  \|  \| \| --- \| --- \| --- \| --- \| | \|  \|  \|  \|  \| \| --- \| --- \| --- \| --- \| | \|  \|  \|  \|  \| \| --- \| --- \| --- \| --- \| | \| 0 \| 4 \|  \|  \| \| --- \| --- \| --- \| --- \| | \| 0 \| 2 \|  \|  \| \| --- \| --- \| --- \| --- \| | \|  \|  \|  \|  \| \| --- \| --- \| --- \| --- \| | \|  \|  \|  \|  \| \| --- \| --- \| --- \| --- \| | \| 1 \| 3 \|  \|  \| \| --- \| --- \| --- \| --- \| | \| 5 \|  \|  \|  \| \| --- \| --- \| --- \| --- \| | \| 4 \|  \|  \|  \| \| --- \| --- \| --- \| --- \| | \|  \|  \|  \|  \| \| --- \| --- \| --- \| --- \| | \| 4 \|  \|  \|  \| \| --- \| --- \| --- \| --- \| | \| 7 \|  \|  \|  \| \| --- \| --- \| --- \| --- \| | \| 4 \|  \|  \|  \| \| --- \| --- \| --- \| --- \| | \| 3 \| 4 \|  \|  \| \| --- \| --- \| --- \| --- \| | \|  \|  \|  \|  \| \| --- \| --- \| --- \| --- \| | \| 6 \|  \|  \|  \| \| --- \| --- \| --- \| --- \| | \| 2 \|  \|  \|  \| \| --- \| --- \| --- \| --- \| | \| 3 \|  \|  \|  \| \| --- \| --- \| --- \| --- \| | \| 0 \|  \|  \|  \| \| --- \| --- \| --- \| --- \| | \|  \|  \|  \|  \| \| --- \| --- \| --- \| --- \| | \|  \|  \|  \|  \| \| --- \| --- \| --- \| --- \| |

The changes in the transcription binding sites of understudied HPV-16 isolates compared with the reference NC_001526.4.

| 0= [YY1](http://alggen.lsi.upc.es/cgi-bin/promo_v3/promo/promo.cgi?dirDB=TF_8.3&idCon=157683706900&getFile=factors/0.html) | 1= TFIID | 2= ROX1 | 3= POU2F2 (Oct-2.1) | 4= NF-1 | 5= E2F | 6= AP-1 | 7= POU2F1 |
| --- | --- | --- | --- | --- | --- | --- | --- |
